# Action or Stimulus: Individual Beliefs About Learned Associations Influence the Processing of Immediate and Delayed Feedback

**DOI:** 10.1111/ejn.70451

**Published:** 2026-03-06

**Authors:** Christine Albrecht, Marta Ghio, Christian Bellebaum

**Affiliations:** ^1^ Faculty of Mathematics and Natural Sciences Heinrich Heine University Düsseldorf Düsseldorf Germany

**Keywords:** feedback delay, feedback‐association types, FRN/N2, N170, prediction error

## Abstract

Feedback learning seems to involve two systems, the striatal reward system and the medial temporal lobe (MTL), which have both been linked to event‐related potential (ERP) components such as the feedback‐related negativity (FRN)/N2, overlapped by a reward positivity (RewP), and the N170, respectively. In this study, we tested the hypothesis that the former system is more involved in associating the feedback with previous actions and the latter in associating the feedback with previous stimuli. More specifically, we hypothesized that the engagement of these systems depends on individual beliefs in credit assignment, that is, whether participants linked the feedback they received to actions or stimuli, possibly modulated by feedback timing. Electroencephalography (EEG) data were recorded from 43 participants performing an ambiguous feedback–learning task, in which feedback could be attributed to either a performed action or a selected stimulus, according to the instruction. As revealed by an Action Index derived from behavioral data, the focus on stimulus–feedback associations was generally stronger than that on action–feedback associations. We found that both FRN/N2 and N170 were influenced by individual beliefs about learned associations, with the FRN/N2 showing stronger feedback valence coding across feedback delays when participants took action–feedback associations into account. Also prediction error coding in the N170 was more pronounced for stronger action–feedback association learning. The results seem to suggest that both learning systems are recruited, at least to some extent, when action–feedback and stimulus–feedback associations are considered simultaneously.

## Graphical abstract

Individual beliefs on whether feedback refers to a previous action or a previous stimulus affects feedback processing: The feedback‐related negativity, possibly indicating striatal activity, showed stronger feedback valence coding across feedback delays, while the N170, possibly indicating medial temporal lobe activity, showed stronger prediction error by valence coding when participants focused on action–feedback associations. Besides feedback delay, beliefs about association type thus might influence which learning system is recruited in feedback learning.

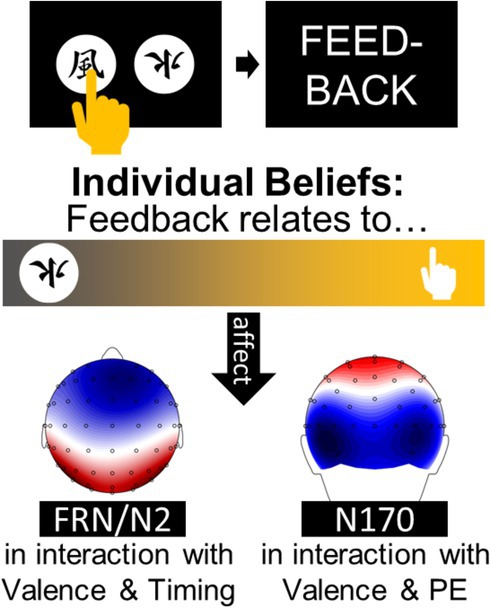

**Abbreviations**
EEG: electroencephalography
ERP: event‐related potential
FRN: feedback‐related negativity
GLME: generalized linear mixed effect
LME: linear mixed effect
MTL: medial temporal lobe
PE: prediction error
RewP: reward positivity

## 1 Introduction

The ability to learn from feedback has primarily been linked to the dopaminergic reward system (Holroyd and Coles 2002) and the striatum (Marco‐Pallarés et al. 2007; Vassiliadis et al. 2024; see Daniel and Pollmann 2014 for a review). The striatum projects to (and receives projections from) the anterior cingulate cortex (Chau et al. 2018; Hauser et al. 2014; see Holroyd and Coles 2002), which has been identified as the neural generator of an event‐related potential (ERP) component in response to feedback. The component appeared as a frontocentral negative deflection occurring about 250 ms after feedback presentation (Miltner et al. 1997; Yeung et al. 2005). It was thus termed feedback‐related negativity (FRN; Gehring and Willoughby 2002; Hauser et al. 2014; Nieuwenhuis et al. 2004). However, its exact nature remains unclear: While the more negative deflection after negative compared to positive feedback (Hajcak et al. 2006; Miltner et al. 1997) was initially interpreted as an indicator of negative feedback processing, more recent research suggests that the difference is rather caused by a slow‐wave, overlaying positive deflection following positive feedback, termed reward positivity (RewP; Proudfit 2015). In line with this, principal component analysis (Foti et al. 2011) revealed an N2‐like component that was identical for rewards and non‐rewards, and a positivity that occurred only for rewards and reduced the amplitude of the negative component in the combined signal. Both striatal activity (Carvalheiro et al. 2021; Diederen et al. 2017; Schönberg et al. 2007) and the ERP amplitude in the N2 time window (Burnside et al. 2019; Fischer and Ullsperger 2013; Röhlinger, Albrecht, and Bellebaum 2025; Röhlinger, Albrecht, Ghio, and Bellebaum 2025; Sambrook and Goslin 2015; Weber and Bellebaum 2024) reflect a reward prediction error (PE), highlighting the role of the dopamine system in feedback processing, as single dopamine neurons also show PE‐related activity (Schultz et al. 1997; Zaghloul et al. 2009). Some studies show that such expectation/PE effects in the N2 time window are strongly pronounced only for positive feedback (Kirsch et al. 2022; Weber and Bellebaum 2024), with more positive amplitudes the more unexpected the feedback, supporting the notion that the ERP signal is driven by a RewP. However, although expectation effects for negative feedback are less consistent, they have been reported (Hoy et al. 2021; Röhlinger, Albrecht, and Bellebaum 2025), with more negative amplitudes the more unexpected the feedback. Cavanagh and Holroyd (2026) suggest that the RewP is mainly modulated by unexpected positive feedback, while the FRN/N2 is mainly modulated by unexpected negative feedback. As the components driving the ERP amplitude in the N2 time window remain unclear, and we are measuring the peak‐to‐peak signal, we refer to the signal as FRN/N2 throughout this manuscript. We expect the peak‐to‐peak measure to mainly reflect an FRN, although the signal might be further modulated by an overlapping RewP.

Evidence suggests that feedback learning can also involve the medial temporal lobe (MTL), specifically the hippocampus (Dickerson and Delgado 2015; Dickerson et al. 2011). This involvement seems to depend on feedback timing (Foerde et al. 2013; Foerde and Shohamy 2011): the striatum is more active in learning from immediate feedback (up to 1 s after the event the feedback refers to), while the MTL is more active in learning from delayed feedback (e.g., about 6 s after the event the feedback refers to). In this context, the MTL may help bridge the temporal gap between event and feedback (DuBrow and Davachi 2016; Staresina et al. 2009). Recent studies suggest that this process may be reflected in the N170 ERP component (Albrecht et al. 2023; Arbel et al. 2017; Höltje and Mecklinger 2020; Kim and Arbel 2019; Röhlinger, Albrecht, and Bellebaum 2025; Röhlinger, Albrecht, Ghio, and Bellebaum 2025), a negative deflection at temporoparietal sites occurring approximately 170 ms after the presentation of feedback. The N170 has traditionally been associated with higher‐order visual processing (Rossion 2014; Rossion et al. 2003) and originates in the fusiform gyrus (Deffke et al. 2007; Gao et al. 2019). In the context of feedback processing, the increased N170 for delayed compared to immediate feedback (e.g., Arbel et al. 2017; Höltje and Mecklinger 2020) may reflect hippocampal activity (as suggested by Arbel et al. 2017). This is in line with recent evidence suggesting that, like the FRN and RewP, also the N170 reflects a PE during feedback processing (Röhlinger, Albrecht, and Bellebaum 2025; Röhlinger, Albrecht, Ghio, and Bellebaum 2025), as PE‐dependent activation patterns have been found in the hippocampus as well (Bein et al. 2020; Dickerson et al. 2011; Sinclair et al. 2021).

Feedback delay influences how strongly the striatum and MTL contribute to feedback processing, but an open question is whether the type of association learned via feedback also shapes the recruitment of these systems, possibly in interaction with feedback timing. On the one hand, the striatum might be particularly suited for learning associations between actions (as opposed to stimuli) and feedback. This hypothesis rests on two lines of evidence. First, the striatum is involved in action selection and inhibition (Aubert et al. 2000; Calabresi et al. 2014) and specifically in motor learning (Nikooyan and Ahmed 2015; Vassiliadis et al. 2024; Wessel et al. 2023). Second, parts of the dopaminergic reward system are more strongly involved in feedback processing when feedback is given for an own choice action (Bellebaum and Colosio 2014; Bellebaum et al. 2012; Cooper et al. 2012; Kobza et al. 2012; O'Doherty et al. 2004; Yeung et al. 2005). On the other hand, given that the N170 reflects higher‐order visual processing (Rossion 2014; Rossion et al. 2003), it may reflect a reactivation of visual areas in the fusiform gyrus representing the stimulus associated with reward (Röhlinger, Albrecht, and Bellebaum 2025), possibly modulated by the hippocampus (Foerde et al. 2013; Foerde and Shohamy 2011; Lighthall et al. 2018). Such a mechanism could help bind reward outcomes to preceding stimuli (Singer and Frank 2009). For instance, reactivation of the primary somatosensory cortex was observed at reward delivery in a somatosensory discrimination task (Pleger et al. 2008; Pleger et al. 2009), and stimulus‐specific visual representations were reactivated upon feedback presentation in a task with visual stimuli (Schiffer et al. 2014).

We first addressed the research question about the potential effects of the type of association that is learned on feedback learning and processing in two recent ERP studies (Röhlinger, Albrecht, and Bellebaum 2025; Röhlinger, Albrecht, Ghio, and Bellebaum 2025). Specifically, based on the assumptions that the striatum is more involved in linking actions to feedback, while the MTL is more involved in linking (visual) stimuli to feedback, we tested the hypotheses that the FRN plays a more important role in feedback processing when action–feedback associations are learned (Röhlinger, Albrecht, Ghio, and Bellebaum 2025) and the N170 is more important for learning associations between visual stimuli and feedback (Röhlinger, Albrecht, Ghio, and Bellebaum 2025; Röhlinger, Albrecht, and Bellebaum 2025). Indeed, we found initial evidence that feedback processing in the FRN and the N170 depends on the type of learned association, although the results were only partially in line with our hypotheses. We found that the PE effect on the N170 was more pronounced for stimulus–feedback associations than action–feedback associations (Röhlinger, Albrecht, Ghio, and Bellebaum 2025), and more pronounced for associations between visual stimuli and feedback than auditory stimuli and feedback, especially for delayed feedback and over the right hemisphere (Röhlinger, Albrecht, and Bellebaum 2025). For the signal at the peak of the difference wave between positive and negative feedback, possibly partially reflecting both FRN and RewP, PE processing was more pronounced for action–feedback associations compared to a condition with stimulus–feedback associations without a response requirement, as expected. PE processing was strongest, however, when feedback was associated with stimuli. A potential explanation for this finding is that, in these previous studies, the stimuli had to be actively chosen, which may have created the belief among participants that feedback also depended on their chosen action. Indeed, it can be ambiguous whether feedback refers to stimulus or response, a phenomenon related to the credit assignment problem (see, e.g., Bogacz et al. 2007; Dam et al. 2013; Schiffer et al. 2014; for a review, see Rubin et al. 2021).

In the present study, we investigated how far feedback processing depends on participants' individual beliefs on whether a feedback stimulus refers to an action or a visual stimulus. We also examined whether feedback timing modulates this effect. The current study thus aims to expand upon previous findings by having all participants perform the same feedback–learning task, with instructions designed to modulate their tendency to learn either action–feedback or stimulus–feedback associations. We hypothesized the difference between positive and negative feedback, as well as PE processing in the FRN/N2, to be most pronounced in immediate feedback and for participants who attribute feedback to a preceding action. In contrast, we expected the N170 amplitude, as well as PE processing in the N170, to be most pronounced for participants who attribute feedback to a preceding stimulus, especially for delayed feedback.

## 2 Method

The study was preregistered at https://doi.org/10.17605/OSF.IO/Z9MPA. Changes from the procedure described in the preregistration will be pointed out in this section.

### 2.1 Participants

Previous studies showed that 20 participants per group sufficed to reveal feedback and PE processing differences in feedback‐related ERPs (Bellebaum and Colosio 2014; Burnside et al. 2019). Therefore, we aimed at overall 40 participants, distributed in two groups (i.e., the stimulus–instruction group and the action–instruction group, see below). Assuming a dropout rate of 20%, we acquired 50 participants, as determined in the preregistration.

We excluded seven participants overall: one because the instructions were not followed correctly; three because of accuracy rates below 55% in the feedback learning task; one because of more than 5% of missed responses; two because of alpha activity in the EEG data. The remaining 43 participants were between 19 and 37 years old (M = 25.1 years, SD = 4.7 years; 29 women, 14 men). All had normal or corrected‐to‐normal vision and normal hearing abilities, no neurological or psychological illnesses and did not take any medication affecting the central nervous system. Two participants were ambidextrous, 33 right‐handed and 8 left‐handed. The groups consisted of 22 participants in the stimulus–instruction group (19–37 years, M = 26.2 years, SD = 5.5 years; 13 women, 9 men) and 21 participants in the action–instruction group (19–30 years, M = 23.9 years, SD = 3.4 years, 16 women, 5 men, see below for details concerning the groups). The study complied with the declaration of Helsinki and was approved by the ethics committee of the Faculty of Mathematics and Natural Sciences at Heinrich‐Heine‐University, Düsseldorf.

### 2.2 Experimental Task

The experimental task was an adapted version of Röhlinger, Albrecht, Ghio, and Bellebaum (2025) and included six learning sessions, each with four blocks. One block always contained one learning part of 20 trials, followed by a test part of 20 trials. In each trial of the learning parts, participants were asked to choose between two actions/stimuli and received either positive or negative monetary feedback, that is, +4 ct versus −2 ct in Euro (€) currency. Participants received a baseline amount of 7 € and could maximize their reward during the task, which would be paid to them after the completion of the study. Participants were instructed that they could realistically achieve a reward between 17 and 25 €. After completing the task, they learned that their reward was rounded up to 25 €, ensuring equal reimbursement for all. We manipulated Feedback Timing within‐subject in different sessions: Participants received either immediate feedback (1 s delay) or delayed feedback (7 s delay) in two learning sessions each. In two other learning sessions, feedback was also delayed, but six regular tones were presented each second of the delay to enable similar temporal predictability of delayed feedback as for immediate feedback (Kimura and Kimura 2016). As in Röhlinger, Albrecht, Ghio, et al., this resulted in three Feedback Timing conditions: immediate, delayed without tone, and delayed with tone. The Feedback Timing was always constant within one learning session, and the order of the conditions across learning sessions was the same for the first three and last three sessions (e.g., ABCABC) and counterbalanced across participants. Participants were instructed that learning started anew in each learning session.

In our previous study (Röhlinger, Albrecht, Ghio, and Bellebaum 2025), one group of participants learned associations between actions and feedback and another group associations between stimuli and feedback. In this study, however, all participants engaged in the same learning task that involved both stimuli and actions. The associations between stimuli and actions, on the one hand, and feedback, on the other hand, were ambiguous. We manipulated the instructions to have participants focus on either action–feedback or stimulus–feedback associations. To achieve ambiguity, reward probabilities depending on the stimuli and on actions were the same. We used two stimuli in each learning session that could either be presented such that Stimulus A was on the left and Stimulus B on the right, or the other way around. For one constellation (e.g., Stimulus A on the left), a specific action (e.g., a left button press) led to reward in 80% of the trials and to punishment in 20% (probabilities were reversed for the other button press). For the other constellation (e.g., Stimulus A on the right), however, reward and punishment probabilities were 50% for both actions. This design created ambiguous contingencies: participants could learn that either a particular action or a particular stimulus led to reward more often than the alternative choice. The respective probabilities for reward and punishment then were 65% versus 35% for both stimulus‐dependent and action‐dependent learning (as in Röhlinger, Albrecht, Ghio, and Bellebaum 2025). For the example provided above, participants could either learn that a left button press was more often rewarded than a right button press or that Stimulus A was more often rewarded than stimulus B. Participants in the action–instruction group were instructed that rewards and punishments were dependent on their action (right or left button press), while participants in the stimulus–instruction group were instructed that rewards and punishments were dependent on the stimulus they had chosen (A or B). Action–instruction participants were told that the stimuli were irrelevant for learning. In contrast, stimulus–instruction participants were instructed that the side on which the stimuli appeared, and thus the chosen action, was of no relevance.

We used 12 Hiragana characters as stimuli in the experiment (see Röhlinger, Albrecht, and Bellebaum 2025). These were randomly chosen from a set of 12 stimuli for each participant, so that two stimuli were assigned to each learning session. It was also randomly determined which of the stimuli was more often rewarded than the other. In three random learning sessions, the correct stimulus was rewarded more often when it was presented on the right, and in the other three learning sessions, when it was presented on the left.

**FIGURE 1:**
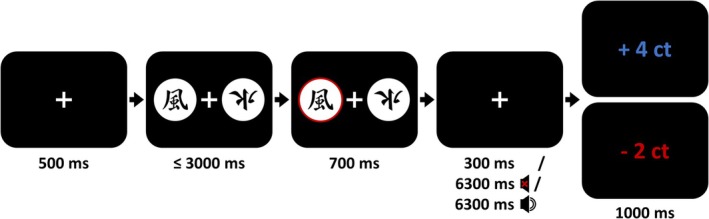
Trial structure in the learning parts.

Figure 1 shows the trial sequence. Each experimental trial in the learning parts started with a 500 ms fixation cross that we instructed participants to focus on. Subsequently, two stimuli appeared on the screen, one on each side of a centrally presented fixation cross (screen side was counterbalanced). Participants had a maximum of 3000 ms to press a right or left button as a response. After the button press, the respective stimulus was highlighted for 700 ms before disappearing, leaving only the fixation cross visible on the screen. Depending on the Feedback Timing condition, the fixation cross stayed on screen for 300 ms (immediate feedback condition) or 6300 ms (two delayed feedback conditions). In the delayed feedback without tone condition, participants saw only the fixation cross during the delay. In the delayed feedback with tone condition, participants additionally heard six 800 Hz tones that were 700 ms long. The first tone started exactly 1 s after the choice (or 300 ms after the highlighted stimulus disappeared), and the following tones each 1 s after the previous tone onset. After the delay, feedback (+4 ct or −2 ct in either blue or red, respectively) was presented for 1000 ms. If participants failed to answer within 3000 ms, they immediately received a note to answer more quickly, and the trial was discarded from the data analyses. Trials in the test part matched trials in the learning part, but participants received no feedback in these trials. Instead, the next trial started immediately after the highlighting of the chosen stimulus and the display of a 300 ms fixation cross.

### 2.3 EEG Recording

EEG data were collected using a BrainAmp DC amplifier and Brain Vision Recorder software (BrainProducts, Germany) with 60 scalp electrodes affixed with an actiCap textile softcap (BrainProducts, Germany). The electrodes were distributed according to the extended 10–20 system and were placed at the following scalp locations: AF3, AF4, AF7, AF8, C1, C2, C3, C4, C5, C6, CP1, CP2, CP3, CP4, CP5, CP6, CPz, Cz, F1, F2, F3, F4, F5, F6, F7, F8, FC1, FC2, FC3, FC4, FC5, FC6, FT10, FT7, FT8, FT9, Fz, O1, O2, Oz, P1, P2, P3, P4, P5, P6, P7, P8, PO10, PO3, PO4, PO7, PO8, PO9, POz, Pz, T7, T8, TP7, and TP8. The online reference was recorded at FCz, while the ground electrode was placed at AFz. Two additional electrodes were positioned on the left and right mastoids. Vertical eye movements were recorded with two electrodes (vertical electrooculogram) below and above the left eye. The EEG was recorded at a sampling rate of 1000 Hz using an online low‐pass filter of 100 Hz. Impedances were kept below 15 kΩ.

### 2.4 Procedure

Upon arrival, participants gave written informed consent to participate in the study and completed a demographic questionnaire. EEG electrodes were then attached to the scalp. The following computer experiment was presented on a 1920*1080 px desktop monitor and responses were made on a Cedrus response pad (RB‐740, Cedrus Corporation, San Pedro, CA, USA). The computer experiment itself lasted about 75 min, and, together with EEG preparation, the session lasted about 2.5 h. The experiment was controlled by Presentation Software (Version 22.0, Neurobehavioral Systems, Albany, CA, USA). After completing the experiment, participants received 25 € reimbursement in the week following their participation.

### 2.5 Data Analysis

#### 2.5.1 Behavioral Data Analyses

##### 2.5.1.1 Accuracy

For the behavioral data analyses, we investigated accuracy, and thus learning, based on those trials in which the correct response for stimulus‐based learning was the same as for action‐based learning (e.g., when the stimulus appearing on the left was more often rewarded than the stimulus on the right). Although we preregistered analyses of the test parts only, we decided to also analyze the learning parts for the following reasons: first, participants might respond differently when feedback is available; second, they might already have learned associations in the first learning block, making effects harder to detect in the test blocks alone. Separate analyses were conducted for the learning parts and test parts. Specifically, we conducted two generalized linear mixed effect (GLME) analysis using the lme4 package in R (Bates et al. 2015): Accuracy in the learning parts was set as dependent variable in the model specified for the first analysis, whereas accuracy in the test parts was set as dependent variable in the model specified for the second analysis. In both models, the fixed effect predictors entering the analysis were Instruction (categorical between‐subject; action–instruction group [−0.5], stimulus–instruction group [0.5]) and Feedback Timing (categorical within‐subject; immediate feedback, delayed feedback without tone, and delayed feedback with tone [using a simple coding contrast matrix with immediate feedback as baseline]). To investigate learning effects over time, we also included the factor Block (continuous within‐subject; Blocks 1–4 for each session, scaled to lie between −0.5 and 0.5). We included random intercepts and slopes per participant. Following guidelines for best practice (Meteyard and Davies 2020), we set the highest complex model possible, as long as the inclusion of each random slope did not lead to over‐ or underfitting, using the function buildmer (Version 2.11). This resulted in the following model for both analyses:

$$\begin{array}{c} \text{Accuracy}\sim \text{Instruction}*\text{Feedback Timing}*\text{Block}\\ +\left(1+\text{Feedback Timing}*\text{Block}\;\right|\text{Subject}) \end{array}$$

##### 2.5.1.2 Action Index

We calculated an Action Index for each participant in each block, reflecting the participants' belief whether a specific action or a specific stimulus predicts reward. To calculate the Action Index, we analyzed choices from those test trials in which both responses by the participants led to reward in half of the trials during the learning (e.g., when Stimulus A was presented on the right side in the example provided above). In these trials, the “better action” and the “better stimulus” were dissociated. For example, if a participant had learned that Stimulus A predicted reward more often than B, they would press the right button when Stimulus A was presented on the right side. If, however, a participant had learned that left button presses were rewarded more often than right button presses, they would press the left button also in trials in which Stimulus A was shown on the right. A higher Action Index indicated that participants attributed feedback more to their action, while a lower Action Index indicated that participants attributed feedback more to the chosen stimulus. The Action Index thus reflected to what extent the participants' beliefs concerning associations between actions and feedback, on the one hand, and stimulus and feedback, on the other hand, corresponded to the instruction. According to the preregistration we wanted to use one Action Index per participant, across all blocks within‐subject. However, because we observed differences in the Action Index between Feedback Timing conditions and because the Action Index depends on learning success (high and low Action Index values for high learning success, medium values for low learning success), we decided to calculate the Action Index for each block. To check for differences in Action Index between the Instruction groups, but also between Feedback Timing conditions and across the Blocks, we specified a model containing the Action Index as dependent variable and the same fixed effects as in the analyses of accuracy (see above). The random effect structure was determined according to the procedure described above. This resulted in the model:

$$\text{Action Index}\sim \text{Instruction}*\text{Feedback Timing}*\text{Block}+\left(1\;\right|\text{Subject})$$

##### 2.5.1.3 PE Modelling

We modelled PEs based on learning trial responses using a reinforcement learning model (Rescorla and Wagner 1972; Sutton and Barto 2018) to calculate the PE:

$$Q_{c,t+1}=Q_{c,t}+\alpha *\delta_{c,t}$$

$Q_{c,t}$ is the value of the chosen action/stimulus in a given trial, α is the participant's individual learning rate. The PE ($\delta_{c,t}$) was calculated as follows:

$$\delta_{c,t}=r_{t}-Q_{c,t}$$

The reward $r_{t}$ is 1 for positive and 0 for negative feedback. Defining the best fitting model involves some degrees of freedom (see Burnside et al. 2019; Röhlinger, Albrecht, and Bellebaum 2025; Weber and Bellebaum 2024). To account for that, we tested 16 different model constraints and chose the one with the lowest Akaike information criterion (for a detailed description of all models and their comparisons, see Supporting Information S1: Section S1). The final model was based on both chosen actions and chosen stimuli: For each participant, independent reinforcement learning models were fitted for action–feedback and stimulus–feedback associations. Each of the six action pairs and six stimulus pairs (for six learning sessions) started with a value ($Q_{c,t}$) of 0.5. Values were updated (using the respective reinforcement learning model) in each trial based on the participants' choice and the respective feedback. The value of the unchosen stimulus or action was always 1‐$Q_{c,t}$. The probability p of the combined action and stimulus models selecting the same action/stimulus as the participant was calculated for each trial using a *softmax* function depending on the prior values of both available actions and stimuli combined. The prior values were calculated as a combination of action and stimulus expectations, weighted by the Action Index across all trials per participant (AI in the formula; the overall Action Index weight resulted in better model fit than Action Index per block, see Supporting Information S1: Section S1):

$$\mathrm{Qc},t\;\text{combined}=\mathrm{Qc},t\;\text{action}*\;\mathrm{AI}+\mathrm{Qc},t\;\text{stimulus}*\;\left(1-\mathrm{AI}\right)$$

Qu,tcombined=1−Qc,tcombined

The exploration parameter β reflects the degree to which the prior values affect the participant's choices.

$$p_{c,t}=\frac{e^{\mathrm{Qc},t*\beta }}{e^{\mathrm{Qc},t*\beta }+e^{\mathrm{Qu},t*\beta }}$$

The determined probabilities p were used to calculate the negative summed log‐likelihood $\left(-\mathrm{LL}\right)$ which was used as a measure for the model's goodness of fit:

$$-\Sigma \;\log\left(p_{c,t_{1},\ldots ,n_{\text{trials}}}\right)$$

The final model included two different learning rates for positive and negative feedback, meaning that the model allowed participants to differ in how well they learned from positive compared to negative feedback. For trials with positive feedback, the learning rate $\alpha_{\mathrm{pos}}$ was used, for trials with negative feedback, the learning rate $\alpha_{\mathrm{neg}}$ was used.

We used MATLAB's *fmincon* function to estimate the free parameter values ($\alpha_{\mathrm{con}}$, $\alpha_{\mathrm{dis}}$, β) for the reinforcement level that best matched the model's predictions to participant behavior by minimizing −LL. Parameters were constrained to [0; 1] for $\alpha_{\mathrm{con}}$ and $\alpha_{\mathrm{dis}}$ and [0; 100] for β. To avoid local minima, we ran the fitting process 50 times with random starting values (within their constraints) for the three free parameters. After determining the best‐fitting free parameters per participant, the parameters were used to calculate action and stimulus values in each trial for the respective participant, using independent reinforcement learning models, and combined PE values dependent on the action and stimulus values:

$$\delta_{c,t}=r_{t}-\left(Q_{c,t\;\text{action}}*\mathrm{AI}+Q_{c,t\;\text{stimulus}}*\left(1-\mathrm{AI}\right)\right)$$

Please note that separate PE values for stimulus and action were used to update action and stimulus values in each trial, but the combined PE was used as a measure of expectancy for the FRN/N2 and N170 analyses. The model results in PE values between −1 (unexpected negative feedback) and 1 (unexpected positive feedback). To dissociate feedback valence and expectancy, we used the unsigned PE (absolute values) and Feedback Valence as separate predictors in the later EEG analyses (see Röhlinger, Albrecht, and Bellebaum 2025; Röhlinger, Albrecht, Ghio, and Bellebaum 2025; Weber and Bellebaum 2024). For an investigation of learning rates (α values) between instruction groups, and an exploration of expected values and PE across the trials of each learning session, see Supporting Information S1: Section S2.

#### 2.5.2 EEG Data Analyses

##### 2.5.2.1 Preprocessing

EEG data preprocessing was performed using BrainVision Analyzer 2.2 (Brain Products GmbH, 2018). The data were re‐referenced to the average of all scalp electrodes (leaving out the mastoids because of bad data quality of the left mastoid electrode), then filtered with a 30 Hz low‐pass and 0.1 Hz high‐pass filter. We used independent component analysis (ICA) and reverse ICA to remove horizontal eye movement artifacts by identifying and removing a blink‐related component. Segments were created from 200 ms before to 800 ms after feedback onset, baseline‐corrected on the basis of the 200 ms before the feedback, and those with artifacts were automatically removed (i.e., all segments with voltage steps > 50 μV/ms, differences between values of > 100 μV or < 0.1 μV within an interval of 100 ms or total amplitudes > 80 μV or < −80 μV). We computed averages across segments for each feedback condition (negative or positive for immediate, delayed without tone, delayed with tone), resulting in six averages per participant. Averages and single‐trial segments were exported for further analyses in MATLAB R2021a (The MathWorks Inc.).

Although we originally planned to derive single trial ERP amplitudes based on the negative feedback‐positive feedback difference wave, similar to our previous studies (Röhlinger, Albrecht, and Bellebaum 2025; Röhlinger, Albrecht, Ghio, and Bellebaum 2025), visual inspection of the frontocentral ERPs showed that differences between conditions already occurred at and before the P2 component and thus before the FRN/N2 (see Figure 4A). The FRN/N2 has been identified to occur after the P2 (see Foti et al. 2011), and is thus influenced by this component. FRN/N2 calculation based on the difference wave thus seemed inappropriate. Instead, we opted for a peak‐to‐peak measure to assess the FRN/N2 to account for differences of the preceding P2 (e.g., Holroyd et al. 2003; Höltje and Mecklinger 2020; Peterburs et al. 2016). For the FRN/N2 analysis, we used data from a frontocentral cluster of electrodes (Fz, FC1, FCz, FC2, Cz; Weber and Bellebaum 2024). For each feedback timing condition and each participant, we used the average signal to find the maximum negative peak between 200 and 400 ms after feedback onset and the preceding maximum positive peak between 100 ms after feedback onset and the latency of the negative peak (corresponding to the P2). Importantly, we derived these latencies individually by participant and condition. An overview of the mean and distribution of latencies of the FRN/N2 is given in the Supporting Information S1: Table S1. We used these latencies to extract the amplitude values for single‐trial segments: for each segment, we extracted the averaged amplitudes from 10 ms before to 10 ms after the respective negative and positive peak latency for the respective participant and condition. We then subtracted the amplitude corresponding to the positive peak from the amplitude corresponding to the negative peak in each segment.

For the N170 analysis, we used data from electrodes P7 and P8 (as in Röhlinger, Albrecht, and Bellebaum 2025; Röhlinger, Albrecht, Ghio, and Bellebaum 2025). Negative peak latencies between 140 and 250 ms post‐feedback were determined in the averages for each participant, feedback condition and electrode. An overview of the mean and distribution of latencies of the N170 is given in the Supporting Information S1: Table S2. Single‐trial amplitudes were again calculated as the mean signal 10 ms before to 10 ms after the identified participant‐, condition‐ and electrode‐specific peak latency.

##### 2.5.2.2 Statistical Analysis of the ERP Data

The ERP data were analyzed using linear mixed effect (LME) analyses with the lme4‐package in R (Bates et al. 2015). For the FRN/N2, we created a model with single‐trial amplitudes at the pooled signal of Fz, FCz, Cz, FC1, and FC2 as dependent variable (see above). As fixed‐effect predictors, we modelled the Action Index (continuous between‐subject factor, scaled, and mean‐centered), Feedback Timing (categorical within‐subject factor with levels immediate feedback, delayed feedback without tones, and delayed feedback with tones [using a simple coding matrix with immediate feedback as baseline]), Feedback Valence (categorical within‐subject factor with levels negative feedback [−0.5] and positive feedback [0.5]), as well as unsigned PE (continuous within‐subject factor, scaled, and mean‐centered). Because Action Index values differed both between and within subjects, adding random slopes by participant might lead to endogeneity effects (Antonakis et al. 2021). Therefore, we only included random intercepts by participant, resulting in the model:

$$\begin{array}{c} \mathrm{FRN}/\text{N2}\sim \text{Action Index}*\text{Feedback Timing}*\text{Feedback Valence}\\ {}*{\mathrm{PE}}_{\text{absolute}}+\left(1\;\right|\text{Subject}) \end{array}$$

For the N170, we created a model with single‐trial N170 amplitudes at P7 and P8 as dependent variables and the same fixed‐effect predictors as for the FRN/N2 analysis. Again, we did not include random slopes by participant due to possible endogeneity, but random intercepts by participant. Additionally, we added random intercepts per electrode (P7, P8) to account for potential amplitude differences between the hemispheres, resulting in the model:

$$\begin{array}{c} N170\sim \text{Action Index}*\text{Feedback Timing}*\text{Feedback Valence}\\ {}*{\mathrm{PE}}_{\text{absolute}}+\left(1\;\right|\text{Subject})+\left(1|\text{Electrode}\right) \end{array}$$

To determine outliers for both analyses, we first calculated the models based on all data. Subsequently, we calculated residuals and removed all datapoints in which the residuum differed more than 2 standard deviations from the mean. The model was then refitted to the cleaned data. The number of included datapoints in the final model is reported for both analyses in the Supporting Information S1: Tables S7 and S9. As we had hypotheses concerning four‐way interactions for both models and it might be questionable whether our sample size was large enough for finding such an effect, we added Bayes statistics for the four‐way interaction effects, calculated with the brms package in R (Bürkner 2017).

## 3 Results

### 3.1 Behavioral Results

**FIGURE 2:**
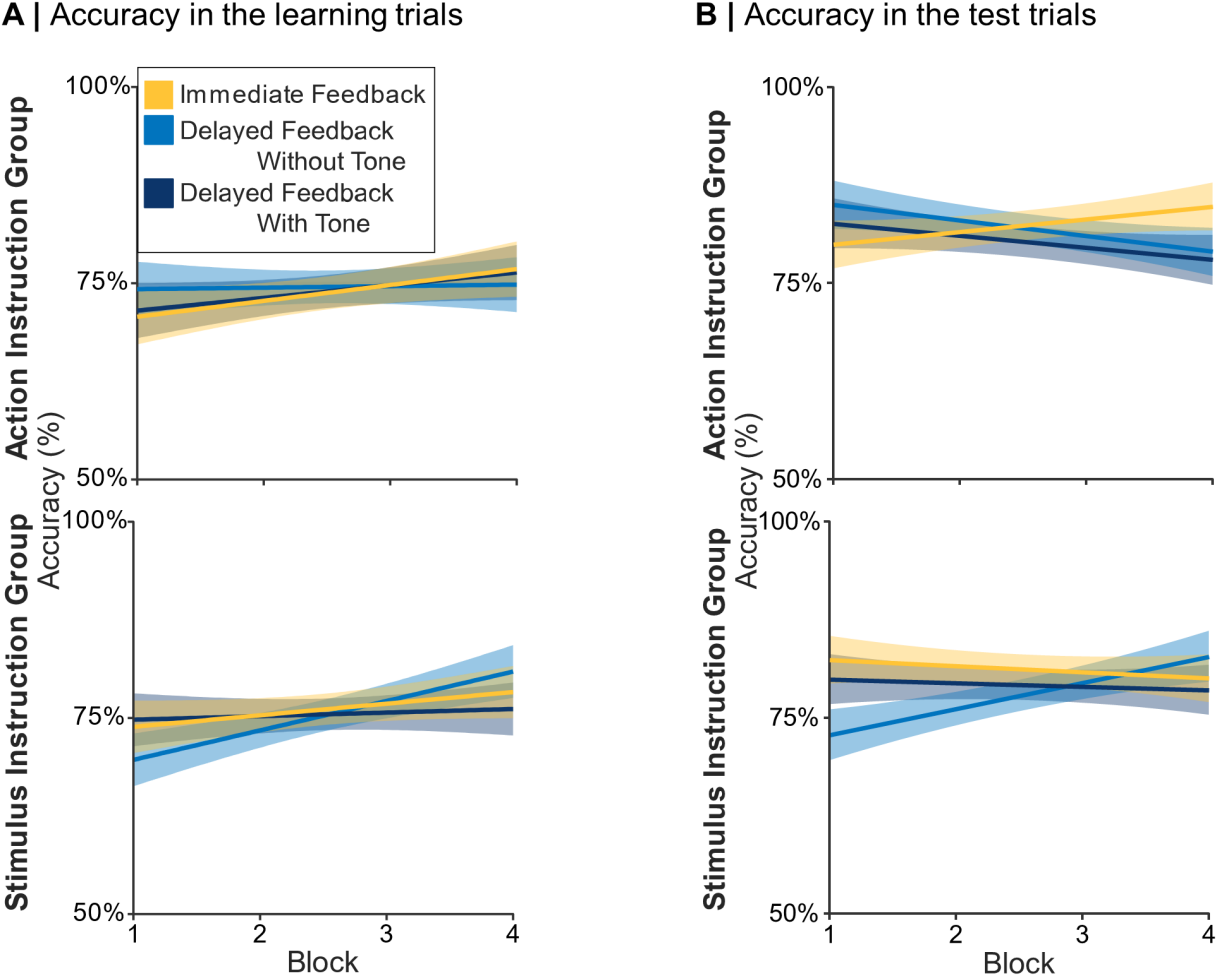
Accuracy in the learning and in the test trials. *Note:* Error margins represent 95% confidence intervals.

For descriptive results concerning accuracy in the learning trials, see Figure 2A. There was a significant main effect of Block, *z* = 2.86, *p* = 0.004, *b* = 0.34. Accuracy increased in later compared to earlier blocks. All other effects were not significant (all *p* ≤ 0.139). For additional statistical results for the GLME analysis of accuracy in the learning trials, including estimates, *z*‐values and confidence interval, see Supporting Information S1: Table S3.

For descriptive results concerning accuracy in the test trials, see Figure 2B. For accuracy in the test trials, we found a three‐way interaction between Instruction, the contrast between immediate feedback and delayed feedback without tones, and Block, *z* = 2.31, *p* = 0.021. Resolving this interaction, there was no significant two‐way interaction between Block and the contrast between immediate feedback and delayed feedback without tones, *z* = −0.86, *p* = 0.388, for the action–instruction group, but for the stimulus–instruction group, *z* = 2.30, *p* = 0.021. For delayed feedback without tones in the stimulus–instruction group, there was a trend effect for Block, *z* = 1.76, *p* = 0.079, *b* = 0.80, and no effect emerged for immediate feedback (*p* = 0.120). No other main or interaction effects were significant (*p* ≥ 0.230). Even though no increase in the number of correct responses over the blocks was found, accuracy was well above chance level in all conditions, indicating that participants learned to choose the more rewarding options, which is consistent with the results of the analysis of accuracy in the learning trials (see above). For additional statistical results for the GLME analysis of accuracy in the test trials, including estimates, *z*‐values and confidence interval, see Supporting Information S1: Table S4.

**FIGURE 3:**
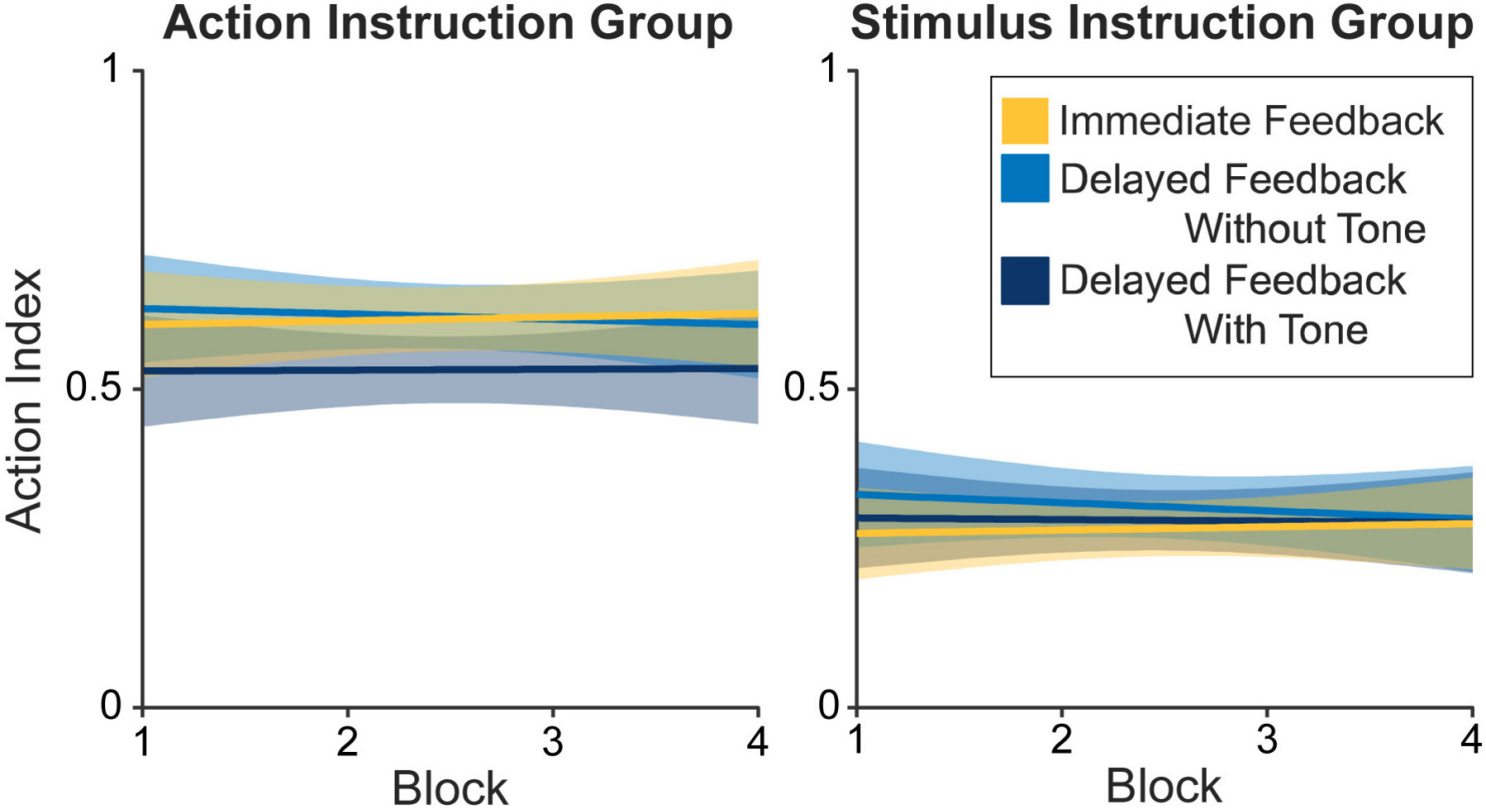
Descriptive Action Index values by Instruction, Block, and Feedback Timing. *Note:* The Action Index codes how much participants attribute feedback to a preceding action: 0 = feedback is attributed to a preceding stimulus; 1 = feedback is attributed to a preceding action. Error margins represent 95% confidence intervals.

For descriptive results concerning the Action Index, see Figure 3. The Action Index differed significantly between the action–instruction and the stimulus–instruction group, *F*(1,41.00) = 26.08, *p* < 0.001, *b* = −0.32, with larger Action Index values for the action–instruction group (M = 0.59, SD = 0.24) than for the stimulus–instruction group (M = 0.28, SD = 0.16). Additionally, the Action Index was dependent on Feedback Timing, *F*(2,979.00) = 3.23, *p* = 0.040, but with no difference either between immediate feedback and delayed feedback without tones (*p* = 0.364) or between immediate feedback and delayed feedback with tones (*p* = 0.109). In an additional contrast, we found a significant difference between the delayed feedback conditions, *t* = −2.51, *p* = 0.012, *b* = −0.06, with lower Action Index values for delayed feedback with tones. All other results were not significant (all *p* ≤ 0.078). For additional statistical results for the GLME analysis of the Action Index, see Supporting Information S1: Table S5.

To further explore Action Index values, we conducted two additional analyses: First, we checked whether variation within‐subjects (between blocks) and between‐subjects (within groups) differed between the action–instruction and stimulus–instruction groups. Second, we analyzed how far Action Index values, or rather, their deviation from 0.5, depended on learning success. These checks are reported in the Supporting Information S1: Section S3. Action Index variation within‐ and between‐subjects was high, but did not differ between Instruction groups. While Action Index values were modulated by learning success, the analysis showed that even for less successful learners, Action Index values differed from chance, making it a valid predictor of individual beliefs.

### 3.2 FRN/N2 Results

**FIGURE 4:**
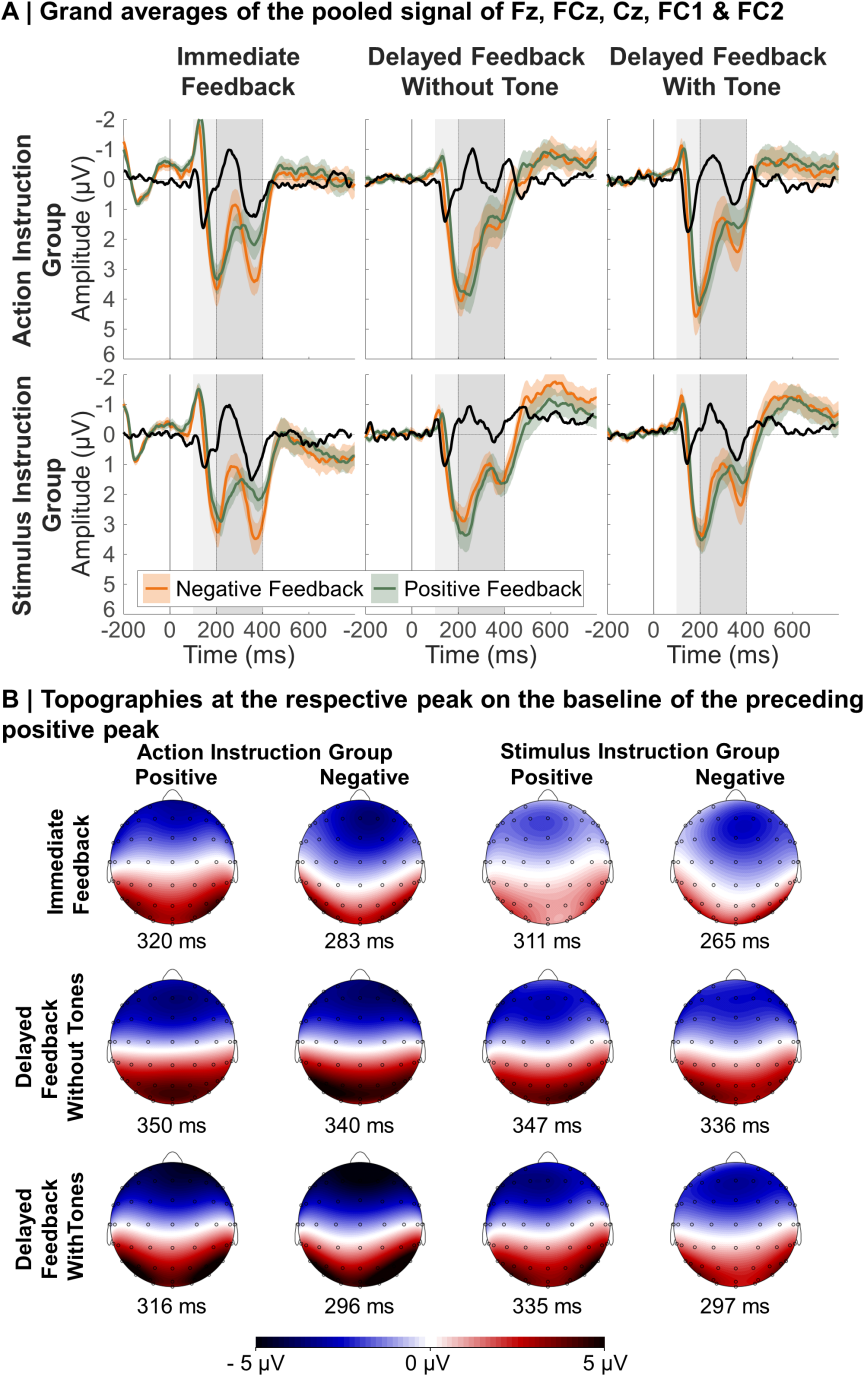
Grand averages and topographies of the FRN/N2. *Note:* (A) Error margins in the event‐related potentials represent standard errors. The black line represents the difference wave. The dark grey rectangle indicates the time window for the extraction of the negative peak; the light grey rectangle indicates the additional time window in which the positive peak was extracted (starting at 100 ms and ending at the latency of the negative peak). (B) The topographies represent the relative negative peak, that is, the negative peak minus the preceding positive peak. For optimal visibility of frontocentral electrodes, the top view of the head is displayed.

**FIGURE 5:**
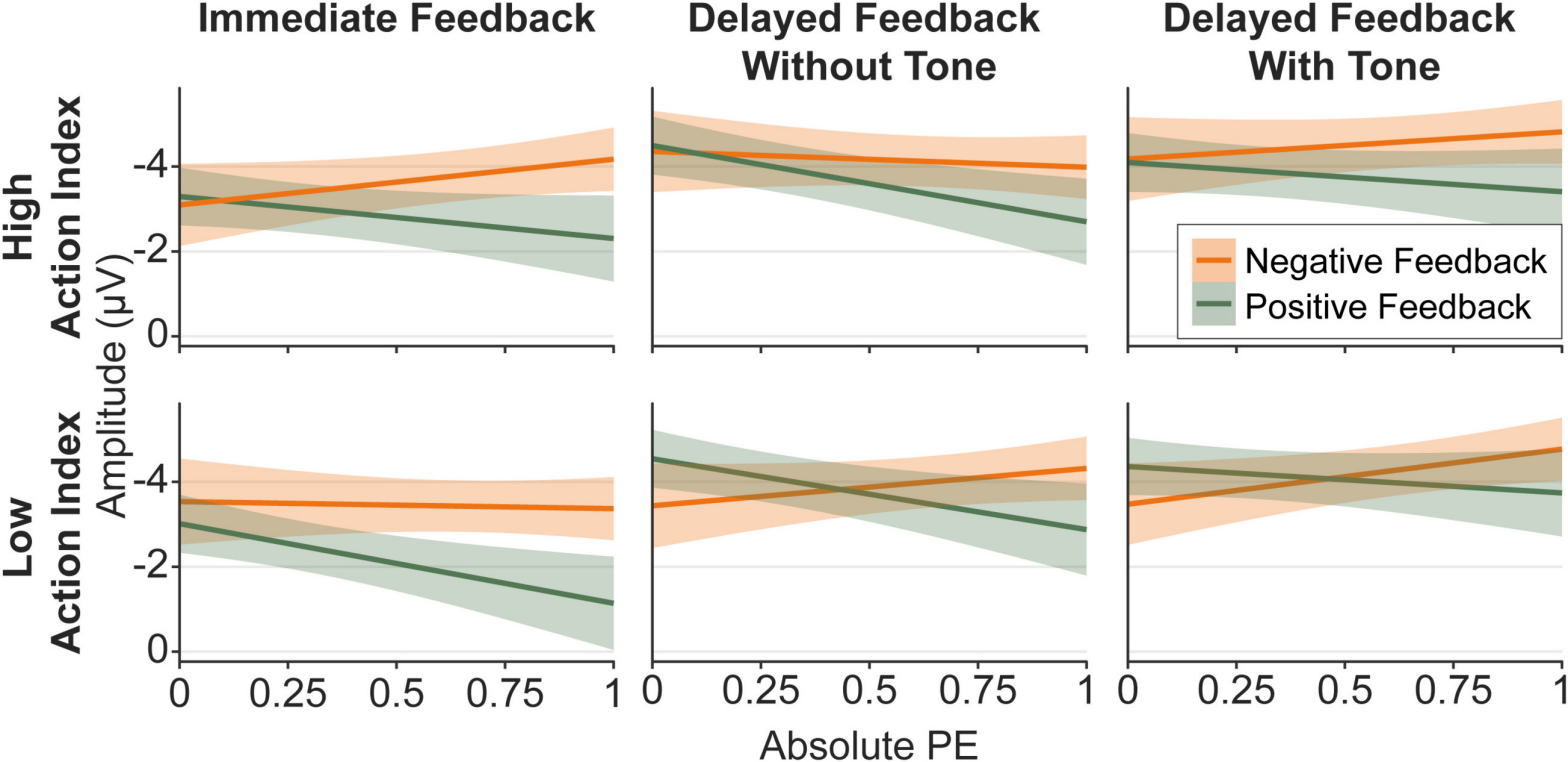
Model predictions of the FRN/N2 model including Action Index. *Note:* Error margins represent 95% confidence intervals. PE = prediction error.

The grand averages and topographies of the FRN/N2 are displayed in Figure 4. For descriptive model statistics, see Figure 5.

Results concerning the effects of Feedback Valence, Feedback Timing, and PE on the FRN/N2 replicated findings of previous studies (Arbel et al. 2017; Burnside et al. 2019; Kim and Arbel 2019; Peterburs et al. 2016; Weber and Bellebaum 2024; Weinberg et al. 2012): We found a significant main effect of Feedback Valence, *F*(1,19306.58) = 29.87, *p* < 0.001, *b* = 0.52, with more negative amplitudes after negative feedback. Further, an interaction between Feedback Timing and Feedback Valence was found, *F*(2,19304.92) = 6.28, *p* = 0.002, with a Feedback Valence effect for immediate feedback, *F*(1,19313.61) = 36.27, *p* < 0.001, *b* = 0.99, but no effect for delayed feedback without tones (*p* = 0.135) and delayed feedback with tones (*p* = 0.055). The interaction was present for both contrasts, immediate versus delayed without tone, *t*(19304.75) = 3.18, *p* = 0.001, and immediate versus delayed with tone, *t*(19304.98) = −2.95, *p* = 0.003. We found a significant Feedback Timing main effect, *F*(2,19307.09) = 48.56, *p* < 0.001, with more negative amplitudes for the two delayed feedback conditions compared to immediate (*t* = −7.41, *p* < 0.001, *b* = −0.86 for delayed feedback without tones, *t* = −9.34, *p* < 0.001, *b* = −1.07 for delayed feedback with tones), in accordance with Peterburs et al. (2016). Finally, a PE main effect, *F*(1,19306.19) = 4.00, *p* = 0.046, *b* = 0.36, could be further explained by a significant PE by Feedback Valence interaction, *F*(1,19344.90) = 23.52, *p* < 0.001. Manual simple slopes analyses revealed that for positive feedback, *F*(1,19339.80) = 24.08, *p* < 0.001, *b* = 1.27, amplitudes were significantly more positive for high compared to low PE values. For negative feedback, amplitudes were significantly more negative for high compared to low PE values, *F*(1,19334.34) = 4.61, *p* = 0.032, *b* = −0.56. Resolving the interaction by PE, we found no significant Feedback Valence effect for low PE values (*p* = 0.777), but for high PE values, *F*(1,19336.03) = 59.24, *p* < 0.001, *b* = 0.99, with more negative values for negative compared to positive feedback.

With respect to our hypotheses for the FRN/N2, we expected the Feedback Timing by Feedback Valence interaction described above to be further modulated by the Action Index. Indeed, we found an interaction between Feedback Timing, Feedback Valence, and Action Index, *F*(2,19304.86) = 3.71, *p* = 0.024, that is depicted in Figure 8A. However, contrary to our expectations, simple slope analyses showed that a Feedback Timing by Feedback Valence interaction emerged only for lower Action Index values (i.e., for participants who attributed the feedback more to the preceding stimulus than action), *F*(2,19304.56) = 9.03, *p* < 0.001, and not for higher Action Index values (*p* = 0.764). Further resolving by Feedback Timing, a Feedback Valence effect emerged for lower Action Index values only in immediate feedback, *F*(1,19313.02) = 25.43, *p* < 0.001, *b* = 1.27, with more negative amplitudes for negative feedback, and not in the two delayed feedback conditions (*p* ≥ 0.847). To investigate the pattern for higher Action Index values, we performed further simple slope analyses and found that for higher Action Index values, peaks were more negative for negative than positive feedback in all Feedback Timing conditions (immediate: *p* = 0.002, *b* = 0.71, delayed feedback without tones: *p* = 0.030, *b* = 0.49, delayed feedback with tones: *p* = 0.006, *b* = 0.66).

We expected a four‐way interaction between Action Index, Feedback Timing, Feedback Valence, and PE, which was not significant (*p* = 0.694, Bayes factor < 0.01). We also found no other significant interactions that included Action Index and PE, and no other main and interaction effects (all *p* ≥ 0.089). For additional statistical results of the FRN/N2 model, including *b*‐values and confidence intervals, see Supporting Information S1: Table S6. For information about the included datapoints per subject and condition, see Supporting Information S1: Table S5.

### 3.3 N170 Results

**FIGURE 6:**
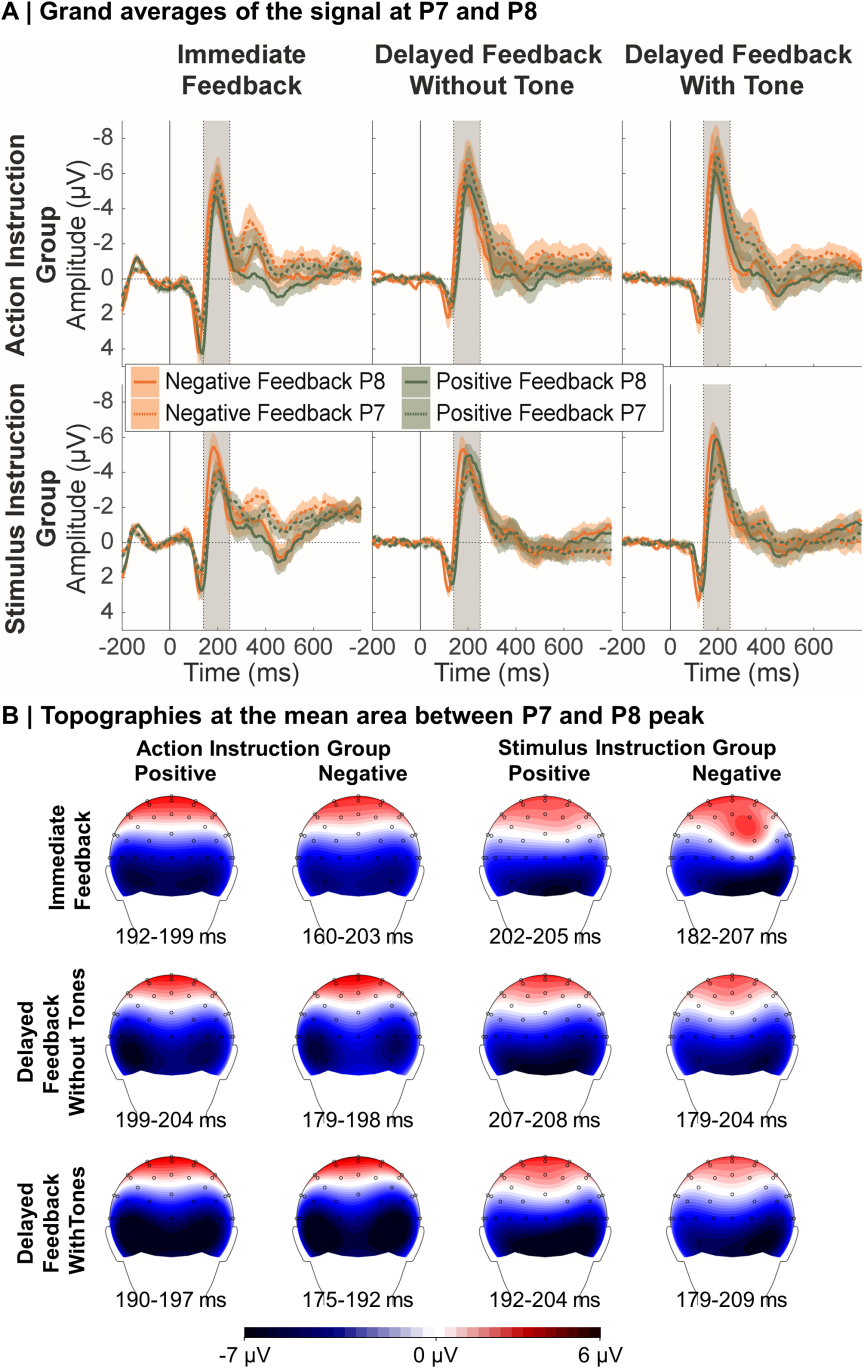
Grand averages and topographies of the N170. *Note:* (A) Error margins in the event‐related potentials represent standard errors. The dark grey rectangle indicates the time window for the extraction of the negative peak. (B) For optimal visibility of temporoparietal electrodes the back view of the head is displayed.

**FIGURE 7:**
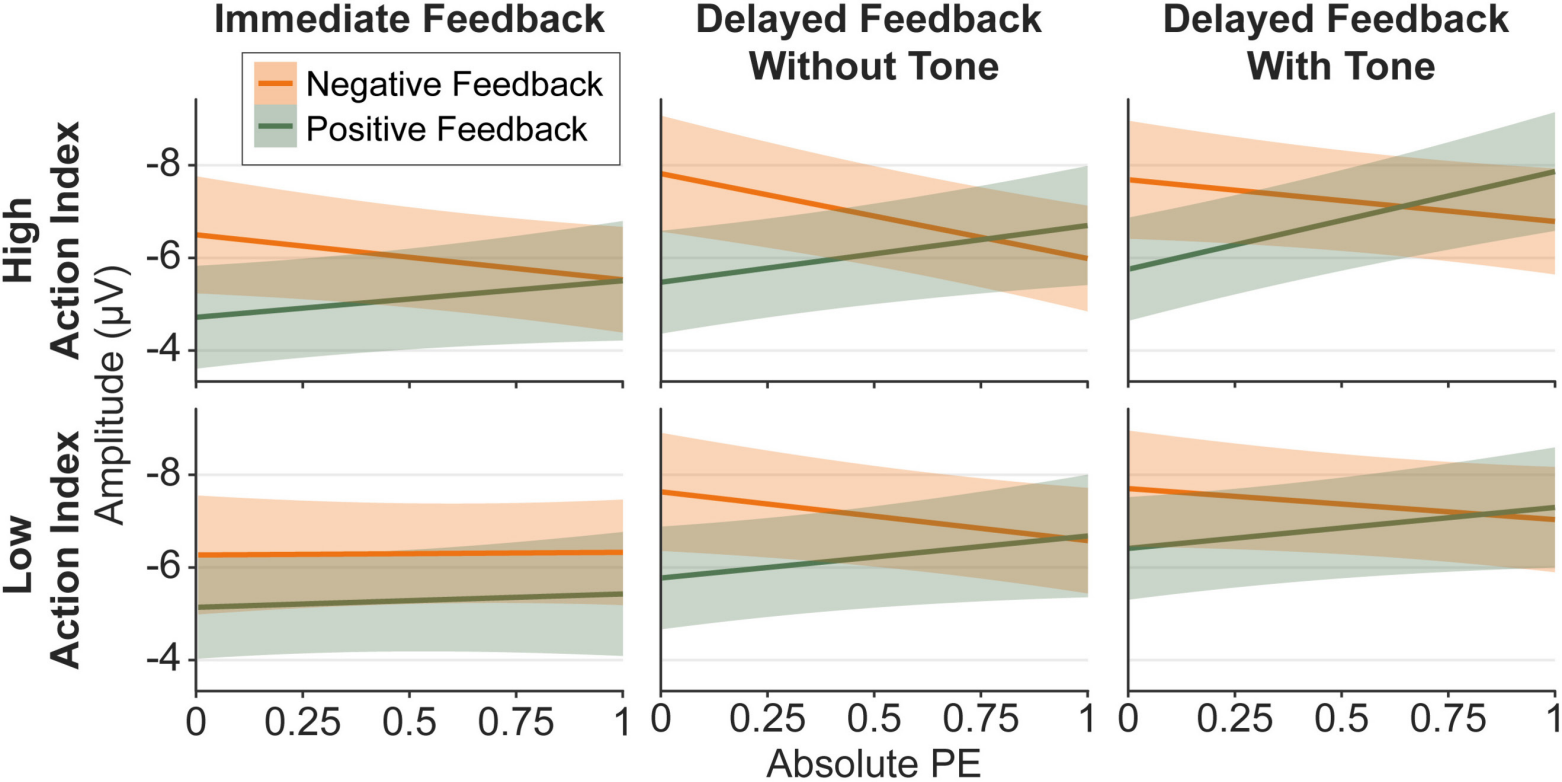
Model predictions of the N170 model including Action Index. *Note:* Error margins represent 95% confidence intervals. PE = prediction error.

The grand averages and topographies of the N170 are displayed in Figure 6. For descriptive model statistics, see Figure 7. As for the FRN/N2, main and interaction results including the factors Feedback Valence, Feedback Timing, and PE replicated results of previous studies (Arbel et al. 2017; Höltje and Mecklinger 2020; Kim and Arbel 2019; Röhlinger, Albrecht, and Bellebaum 2025; Röhlinger, Albrecht, Ghio, and Bellebaum 2025): we found a significant main effect of Feedback Valence, *F*(1,38631.46) = 114.73, *p* < 0.001, *b* = 0.88, with more negative amplitudes for negative compared to positive feedback, and a significant main effect of Feedback Timing, *F*(2,38631.56) = 97.52, *p* < 0.001, with larger (more negative) amplitudes for the two delayed feedback conditions compared to the immediate feedback condition, *t* = −9.11, *p* = < 0.001, *b* = −0.92 for delayed feedback without tones, *t* = −13.73, *p* = < 0.001, *b* = −1.37 for delayed feedback with tones.

Replicating very recent findings from our group (Röhlinger, Albrecht, and Bellebaum 2025; Röhlinger, Albrecht, Ghio, and Bellebaum 2025), we found a significant interaction between Feedback Valence and PE, *F*(1,38657.84) = 34.66, *p* < 0.001. Simple slope analyses revealed that a PE effect emerged for negative feedback, *F*(1,38645.89) = 15.72, *p* < 0.001, *b* = 0.90, with more negative amplitudes for lower compared to higher PE values, and for positive feedback, *F*(1,38650.08) = 21.15, *p* < 0.001, *b* = −1.03, with more positive amplitudes for lower compared to higher PE values. Resolving the interaction by PE, amplitudes were more negative for negative than positive feedback for low PE values, *F*(1,38648.23) = 123.22, *p* < 0.001, *b* = 1.38, and high PE values, *F*(1,38647.33) = 11.31, *p* = 0.001, *b* = 0.38, with a larger effect for low PE values.

**FIGURE 8:**
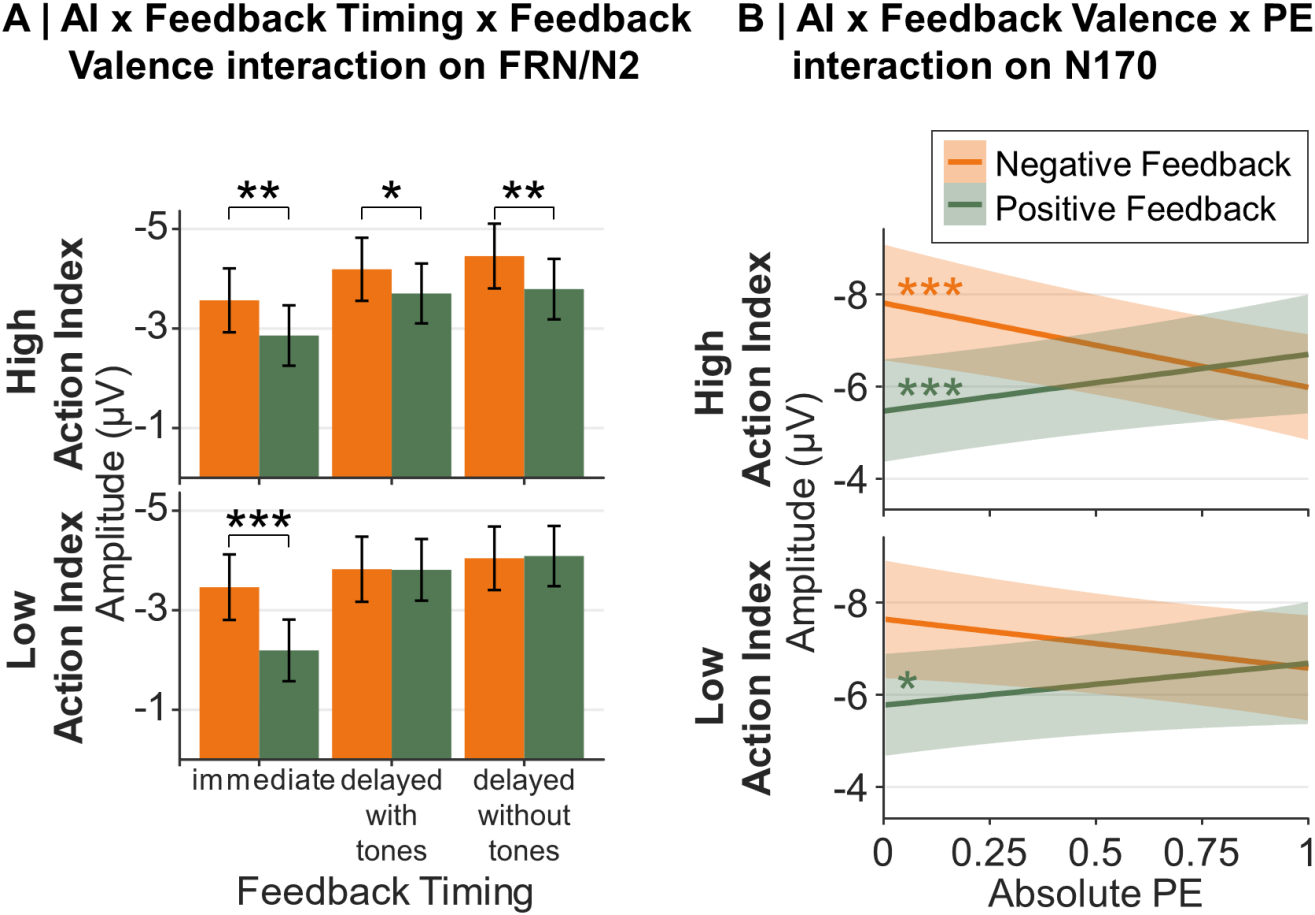
Model predictions of all significant effects involving the Action Index. *Note:* Error margins represent 95% confidence intervals. AI = Action Index, PE = prediction error.

Concerning our hypotheses for the N170, we expected the Feedback Valence and Feedback Timing effects to be further modulated by Action Index, and expected a four‐way interaction between Feedback Valence, Feedback Timing, PE, and Action Index. However, this was not significant (*p* = 0.954, Bayes factor < 0.01) and we only found a significant three‐way interaction between Action Index, Feedback Valence, and PE, *F*(1,38654.51) = 4.55, *p* = 0.033. The two‐way interaction between Feedback Valence and PE was stronger for higher Action Index values, *F*(1,38655.52) = 33.53, *p* < 0.001, than lower Action Index values, *F*(1,38658.83) = 7.33, *p* = 0.007. For higher Action Index values, a significant effect of PE emerged for negative feedback, *F*(1,38641.40) = 15.39, *p* < 0.001, *b* = 1.23 (low PE = more negative) and positive feedback, *F*(1,38650.39) = 19.80, *p* < 0.001, *b* = −1.37 (low PE = more positive). For lower Action Index values, a PE effect emerged only for positive feedback, *F*(1,38650.50) = 4.60, *p* = 0.032, *b* = −0.70 (low PE = more positive), but not for negative feedback (*p* = 0.077). The three‐way interaction is depicted in Figure 8B.

We found no further significant results (all *p* ≥ 0.068). For additional statistical results of the N170 model, including *b*‐values and confidence intervals, see Supporting Information S1: Table S8. For information about the included datapoints per subject and condition, see Supporting Information S1: Table S9.

## 4 Discussion

The present study investigated whether participants engaged different learning systems based on credit assignment for the feedback they received. More specifically, we analyzed in how far feedback processing depended on participants' beliefs about the type of association they were learning, namely, action–feedback or stimulus–feedback associations. Participants completed a feedback–learning task with either immediate or delayed feedback, in which they were instructed to either focus on pressing the preferable (i.e., more rewarding) button (action–instruction group) or selecting the preferable stimulus (stimulus–instruction group). Both groups underwent identical learning trials while EEG was recorded to assess feedback processing. We used ambiguous trials in subsequent test blocks to measure individual beliefs concerning the type of the learned association. We expected that participants with higher Action Index values, indicating a stronger belief in action–feedback associations, would primarily engage the striatal learning system. This would be reflected in pronounced coding of feedback valence and PE in the FRN/N2, probably modulated by the RewP (see below), particularly for immediate feedback. Conversely, participants with a lower Action Index would rely more on the MTL learning system, as reflected in pronounced coding of feedback valence and PE in the N170, particularly for delayed feedback. Bayes analysis of the highest‐order interaction of both EEG models revealed very strong evidence against our hypotheses (Lee and Wagenmakers 2014). These distinctive Bayes factor values suggest that the negative findings were not caused by an insufficient sample size.

Analyses of the Action Index derived from the participants' choice data confirmed that the instructions shaped participants' beliefs concerning credit assignment: Action Index values differed significantly between groups, with higher values in those participants who were told that feedback depended on their chosen action. The stimulus–feedback instruction was slightly more effective, producing Action Index values that deviated more clearly from chance than in the action–feedback instruction group, indicating stronger beliefs. It is important to note that since the Action Index is performance‐dependent—being more extreme in better learners—it is less reliable for interpreting beliefs when learning is poor. However, accuracy in the learning trials was comparable between the two instruction groups and increased across blocks, indicating that learning occurred and did not depend on the belief concerning credit assignment. Also, while additional analyses showed that Action Index was dependent on learning success, the model fit suggested above‐chance level Action Index values also for less successful learners. Although a trend effect of block in the test trials emerged only for the stimulus–feedback instruction in delayed feedback without tones, overall accuracy rates in the test trials were well above chance level and did not differ between conditions. It is likely that associations were formed already after the first learning block in most conditions, explaining the lack of block effects in the test trials. In the delayed feedback without tone condition and stimulus–feedback instructions, learning may have been slower initially, but accuracy descriptively caught up in later blocks. Therefore, differences in Action Index values are best explained by belief differences, not learning performance.

Regarding neural processes linked to learning, we could replicate previous findings that both the FRN/N2 and the N170 reflect a PE. Interestingly, and in accordance with other recent findings from our group (Röhlinger, Albrecht, Ghio, and Bellebaum 2025), the PE by valence interaction for the N170 was driven by stronger valence effects for expected than unexpected events. The FRN/N2 showed a valence effect only for unexpected events, as was also shown before (Kirsch et al. 2022). Cautiously, in line with classical reinforcement learning theories (Holroyd and Coles 2002), the FRN/N2 might signal a need to adapt actions and expectations, which arises when events are unexpected. The N170, in contrast, might code the strengthening of existing associations, possibly reflecting stronger reactivations of the events associated with feedback (e.g., Schiffer et al. 2014). Regarding the underlying components driving the amplitude of the FRN/N2, ERP signals after positive feedback, reflecting a positive PE, might reflect the underlying RewP activity, and ERP signals after negative feedback, reflecting a negative PE, might reflect the FRN (see Cavanagh and Holroyd 2026).

Our first hypothesis was that the difference between positive and negative feedback in the FRN/N2 would be most pronounced for participants who attribute immediate feedback to a preceding action. In line with the hypothesis, we found a respective three‐way interaction between Feedback Valence, Feedback Timing, and the Action Index. But contrary to expectations, valence effects in the FRN/N2 were not particularly pronounced for higher Action Index values in the immediate feedback condition. Instead, a feedback valence effect emerged across all feedback timing conditions for participants who associated feedback with a preceding action (higher Action Index values). Lower Action Index values (when participants attributed feedback to a preceding stimulus) resulted in the typical pattern of reduced valence effects with increasing feedback delay often observed in the FRN or RewP (Albrecht et al. 2023; Höltje and Mecklinger 2020; Peterburs et al. 2016; Weinberg et al. 2012). This pattern of the three‐way interaction, although different from our hypotheses, still aligns with our underlying assumptions: when participants attribute feedback to a preceding action, the tendency to learn via the dopaminergic reward learning system may be stronger (Arbel et al. 2017; Foerde et al. 2013; Foerde and Shohamy 2011; Peterburs et al. 2016; Weinberg et al. 2012), so that participants might have engaged this system irrespective of feedback delay, as reflected in the feedback valence effect across feedback delays. For participants attributing feedback to a stimulus, valence effects were only found for immediate feedback, suggesting that immediate feedback engages the dopaminergic reward system even if the feedback is not linked to a preceding action. Even though drawing direct inferences from ERP components to brain structures is difficult, the data suggest that the proposed role of the striatum for associating feedback with recent events (Jocham et al. 2016; Yagishita et al. 2014) may thus not be limited to recent actions.

Our second hypothesis was that, for the FRN/N2, strongest PE effects, as indicated by the interaction of absolute PE and feedback valence, would emerge if participants attribute feedback to a preceding action for immediate feedback. However, we found no evidence supporting this hypothesis. A significant PE by Valence interaction emerged, showing that more unexpected events elicited more positive amplitudes for positive feedback (see Kirsch et al. 2022; Weber and Bellebaum 2024), but also more negative amplitudes for negative feedback (see Hoy et al. 2021; Röhlinger, Albrecht, and Bellebaum 2025; Röhlinger, Albrecht, Ghio, and Bellebaum 2025), in line with a suggestion by Cavanagh and Holroyd (2026) of positive PE coding in the RewP in response to unexpected goal progression and negative PE coding in the FRN. This interaction was not further modulated by either Action Index or feedback timing. This is in line with our previous studies showing no timing effect on PE coding in the ERP signal in the FRN/N2 time window (Röhlinger, Albrecht, and Bellebaum 2025; Weber and Bellebaum 2024). While null effects are difficult to interpret, Bayesian statistics revealed strong evidence against a four‐way interaction. Together with the previously described three‐way interaction between feedback valence, feedback timing, and Action Index, this pattern of results suggests that valence coding in the FRN/N2 is dependent on participants' belief about the learned associations, but the processing of PEs is not.

Our third hypothesis predicted that the N170 would be most pronounced when participants attributed feedback to a preceding stimulus, particularly for delayed feedback. We found no evidence that supports this hypothesis. Finally, our fourth hypothesis proposed that the N170 would show the strongest PE effect when participants attributed feedback to a preceding stimulus, especially for delayed feedback. Even though we did not find a four‐way interaction between all involved factors, we did find a significant interaction between Feedback Valence, PE, and Action Index. This three‐way interaction further explained an (absolute) PE by valence interaction (indicating PE coding), replicating previous findings from our group (Röhlinger, Albrecht, and Bellebaum 2025; Röhlinger, Albrecht, Ghio, and Bellebaum 2025). The resolution revealed that the PE by valence interaction was more pronounced for higher than lower Action Index values, that is, more pronounced if participants attributed the feedback to an action than to a stimulus, which is the opposite of the hypothesized effect. Combined with our FRN/N2 findings this suggests that the neural systems linked to both ERP components (presumably striatum and MTL‐based learning systems) were more involved when learning depended more strongly on action–feedback associations, independent of feedback timing. Importantly, Action Index values were less extreme (i.e., less clearly deviating from the middle value of 0.5) in participants in the action instruction group than in the stimulus instruction group, indicating a tendency to learn from both, actions and stimuli, in the action instruction group. The result pattern for the two ERP components might thus suggest that both underlying learning systems were recruited for participants with higher versus lower Action Index values. In this sense, the finding is in line with our previous study (Röhlinger, Albrecht, Ghio, and Bellebaum 2025) where we found recruitment of both systems when participants actively chose between different stimuli, possibly linking feedback to stimuli and actions. We initially expected that N170 amplitudes reflected reactivations of brain regions involved in visual processing, particularly for stimulus–feedback associations. This expectation was based on findings that the MTL bridges the temporal gap by binding outcomes to preceding experiences (Singer and Frank 2009) and reactivating associated representations (Pleger et al. 2008; Pleger et al. 2009; Schiffer et al. 2014). This mechanism, however, might also apply to preceding actions. Possibly, in the latter case, the feedback‐locked N170 during learning of action–feedback associations might reflect MTL activity, that could drive the reactivation of motor cortices linked to previously performed actions. Evidence for such reactivation at reward presentation has been found for immediate feedback with short action–feedback intervals (1400 ms), then likely modulated by the striatal system (Cohen and Ranganath 2007). Speculatively, such a reactivation could then allow both learning systems to connect action and feedback.

Assuming that the N170 reflects MTL activity that drives the reactivation of motor cortices in action learning with delayed feedback favors Arbel et al.'s (2017) assumption that N170 directly reflects hippocampal activity. However, our group has found evidence that the N170 encodes PEs more strongly for visual stimulus–feedback associations than for auditory ones, suggesting that the component is linked to the reactivation of visual representations of learned stimuli in the fusiform gyrus (Röhlinger, Albrecht, and Bellebaum 2025). Given the close anatomical proximity of the fusiform gyrus and the hippocampus/MTL, and EEG's poor spatial resolution, the N170 may capture overlapping activity from both regions. It is therefore plausible that its neural source differs between stimulus–feedback and action–feedback learning. A magnetoencephalography study combined with MRI could help pinpoint the exact origin(s) of the N170 in feedback learning contexts.

While thus both learning systems may be recruited in participants with relatively higher Action Index values, the question remains which neural mechanisms underly learning in participants with lower Action Index values. For immediate feedback the striatal reward system appeared to be also involved, as indicated by the pronounced feedback valence effect (see also Höltje and Mecklinger 2020; Peterburs et al. 2016; Weinberg et al. 2012). With respect to the recruitment of the MTL‐based learning system, it is important to note that the N170 reflected the PE also for stimulus–feedback associations, with the restriction that the PE was reflected in N170 amplitude only for positive feedback. For immediate feedback, both learning systems may thus have also cooperated when learning was based on stimulus–feedback associations, while for delayed feedback the focus may have been on MTL‐based learning, especially for positive feedback. As outlined above, the N170 may reflect the strengthening of existing or expected associations (Schiffer et al. 2014), which is particularly important for positive feedback.

As in our previous study (Röhlinger, Albrecht, Ghio, and Bellebaum 2025), our findings contradict the account by Kimura and Kimura (2016), which attributes feedback delay effects solely to reduced temporal predictability. We observed significant differences between immediate feedback and both temporally predictable and temporally unpredictable delayed feedback conditions concerning the effect of feedback valence in the FRN/N2. Since participants in Kimura and Kimura's task could not learn from feedback, we suspect that temporal predictability plays a less central role in feedback learning tasks, where feedback is informative, than in tasks where it cannot be used for learning.

In conclusion, our findings show that individual beliefs about the type of learned associations shape how feedback is processed during learning. The dopaminergic reward system, reflected in the FRN/N2, was more strongly engaged when participants relied more strongly on action–feedback than stimulus–feedback associations, as indicated by robust valence effects regardless of feedback timing. This is consistent with a prominent role of the striatum in linking actions to feedback. At the same time, the N170, possibly reflecting MTL and/or fusiform gyrus activity, showed more prominent PE coding for stronger action–feedback associations. PE coding might be particularly reflected in the N170 if striatum‐ and MTL‐based learning systems cooperate. Especially for action–feedback associations, where modulations of both FRN/N2 and N170 were observed, we speculate that the MTL could help credit assignment in the striatal system by reactivating neural action representations. Future studies using combined imaging approaches could help clarify the neural origins of the N170 in feedback learning and how they vary by association type.

## Author Contributions

**Christine Albrecht:** data curation, formal analysis, investigation, methodology, project administration, software, visualization, writing – original draft preparation. **Marta Ghio:** conceptualization, methodology, project administration, resources, supervision, validation, writing – review and editing. **Christian Bellebaum:** conceptualization, funding acquisition, methodology, project administration, resources, supervision, validation, writing – review and editing.

## Funding

This work was funded by the Deutsche Forschungsgemeinschaft (DFG; German Research Foundation)—Project Number 467460456.

## Conflicts of Interest

The authors declare no conflicts of interest.

## Supporting information


**Data S1:** Supporting information.

## Data Availability

The study was preregistered on 10.17605/OSF.IO/Z9MPA. The data that support the findings of this study and all analysis scripts are openly accessible through the Open Science Framework at 10.17605/OSF.IO/F3R42.
