# Supplementary material for: Action or Stimulus: Individual Beliefs About Learned Associations Influence the Processing of Immediate and Delayed Feedback
Source: Eur J Neurosci. 2026 Mar 6;63(5):e70451. doi: 10.1111/ejn.70451 (PMC12966775; doi:10.1111/ejn.70451)
Supplement: Supplementary file 1 — Data S1: Supporting information. [file EJN-63-0-s001.docx]

**Supplementary Material**

Action or Stimulus: Individual beliefs about learned associations influence the processing of immediate and delayed feedback

Christine Albrecht, Marta Ghio, Christian Bellebaum

Heinrich Heine University Düsseldorf, Faculty of Mathematics and Natural Sciences, Institute of Experimental Psychology, Universitätsstraße 1, 40225, Düsseldorf, Germany

**Section S1**

*Reinforcement learning model comparisons*

To determine the optimal reinforcement learning model for the PE calculation, we compared *-LL* values for a number of models. Because participants could assign feedback to either a previous action or stimulus, and this could affect reinforcement learning models, we tested models for four different assumptions:

A1. Participants always assigned the feedback to the event, action or stimulus, according to the instruction (Instruction Assumption).

A2. If a participant’s overall Action Index (AI) was above or equal to 0.5, they assigned the feedback to an action. If not, they assigned feedback to a stimulus (Split Action Index Assumption).

A3. Participants derived expectations based on both stimulus and action, and weighed them based on the overall Action Index (across all trials) by participant. In the respective models, we calculated reinforcement learning models for each participant both based on stimulus-feedback and action-feedback associations, that were learned independently from each other, but with the same learning rates. To derive the prediction error, we then used the Action Index for each participant as weight and calculated the overall expectation (used to determine the PE for each trial, and used to determine the log likelihood of the model) as follows:

$Q_{t-overall} = Q_{t-action} * {AI}_{subject} + Q_{t-stimulus} * (1-{AI}_{subject})$

A4. Participants derived expectations based on both stimulus and action, and weighed them based on the Action Index of the respective block. We used the same calculation as for A3, but the Action Index by block as weight to calculate the overall expectation in each trial:

$Q_{t-overall} = Q_{t-action} * {AI}_{block} + Q_{t-stimulus} * (1-{AI}_{block})$

For each of the four assumptions, we tested models with four different constraints:

**C_1_.** In the first model, we assigned each action or stimulus pair an initial $Q_{t}$ value of 0.5. The unchosen option was calculated in each trial as 1-$Q_{c,t}$. The model included one learning rate that was used to update values after positive and negative feedback and in each learning session.

**C_2_.** In the second model, we assigned $Q_{t}$ values as for model 1, but allowed for different learning rates for positive and negative feedback.

**C_3_.** In the third model, we again assigned $Q_{t}$ values as in model 1, but allowed one learning rate (used for both positive and negative feedback) for each learning session (six learning rates overall).

**C_4_.** The fourth model assigned $Q_{t}$ values as in model 1, and as in model 3, we allowed different learning rates for each learning session, and additionally different learning rates for positive and negative feedback (12 learning rates overall).

Overall, combining assumptions and constraints, we tested 16 models. The best model fit was achieved for the A_3_C_2_ model, with an Akaike Information Criterion (AIC) value of *AIC* = 428.43 (fits for all models are provided in the Table below). As AIC values were quite high for all C_3_ and C_4_ models, we expected that learning rates differed neither by learning session nor by feedback timing, since feedback timing was modulated across learning sessions.

**Table S1.A**

| *Akaike Information Criterion for all the 16 tested Models***Model** | ***AIC*** |
| --- | --- |
| A_1_C_1_ | 510.88 |
| A_1_C_2_ | 462.85 |
| A_1_C_3_ | 530.14 |
| A_1_C_4_ | 495.48 |
| A_2_C_1_ | 490.71 |
| A_2_C_2_ | 438.42 |
| A_2_C_3_ | 506.57 |
| A_2_C_4_ | 469.22 |
| A_3_C_1_ | 491.05 |
| **A_3_C_2_** | **428.43** |
| A_3_C_3_ | 512.03 |
| A_3_C_4_ | 488.68 |
| A_4_C_1_ | 489.82 |
| A_4_C_2_ | 442.69 |
| A_4_C_3_ | 514.21 |
| A_4_C_4_ | 490.86 |

**Section S2**

*Learning Parameters, Expected Values and Prediction Errors of the Reinforcement Learning Model*

**Alpha Parameters**

To investigate results of the reinforcement learning model, we first compared learning rates (alpha values) across instruction groups and for positive and negative feedback. We used an LME model including Instruction and Feedback Type as fixed effects, and allowed random intercepts by participant. Learning rates were significantly higher for positive than negative feedback, *F*(1,41.00) = 61.61, *p* < .001, *b* = 0.29. Also, Participants in the action instruction group had significantly higher learning rates (alpha values) than participants in the stimulus instruction group, *F*(1,41.00) = 4.98, *p* = .031, *b* = 0.11. There was no interaction between Instruction and Feedback Type (*p* = .733). Descriptive learning rates are reported in Table S2.A.

**Table S2.A**

*Learning Rates (Alpha Values) by Instruction and Feedback Type*

| Instruction | Feedback Type | *M* | *SD* | *Min* | *Max* |
| --- | --- | --- | --- | --- | --- |
| Stimulus | Positive | 0.37 | 0.19 | 0.11 | 0.83 |
|  | Negative | 0.09 | 0.06 | 0.02 | 0.27 |
| Action | Positive | 0.49 | 0.27 | 0.16 | 1.00 |
|  | Negative | 0.19 | 0.22 | 0.02 | 0.87 |

*Note. M* = mean across all participants, *SD* = standard deviation, *Min* = minimum, *Max* = maximum.

**Expected Values**

The reinforcement learning model used to derive PEs assumed that participants updated expected values (Q values) for action pairs and stimulus pairs individually. Therefore, in each trial, the model determined one expected value (Q value) for the action pair and the stimulus pair. For a display of expected values of action and stimulus pairs throughout each learning session, separated by high and low Action Index (Action Index across all trials for one participant, here referred to as overall Action Index), see Figure S2.B. Please note that the expected value is displayed for the preferable action/stimulus; the expected value for the other action/stimulus derives as 1-Q.

**Figure S2.B**

*Expected Values (Q Values) of Action and Stimulus Pairs for Higher and Lower Overall Action Index Values Across Trials of a Learning Session*

*
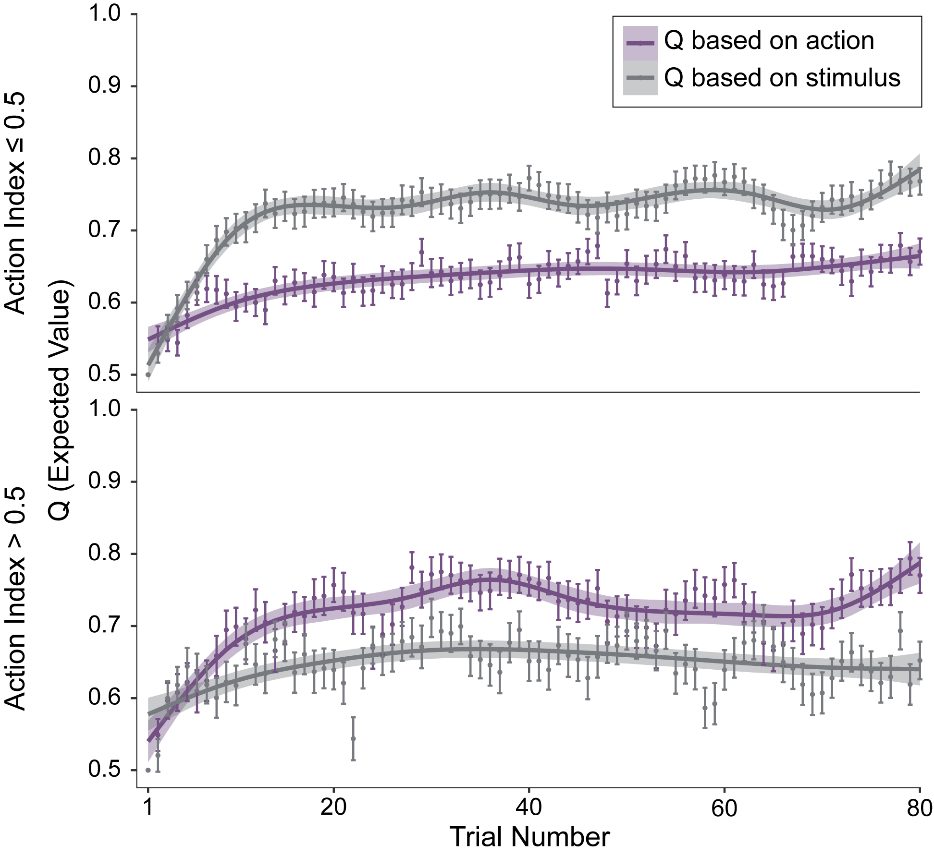
*

To investigate differences in expected (Q) values by overall Action Index (between subject continuous factor, scaled and mean-centred), Pair Type (within-subject categorical factor, Action = 0.5, or Stimulus = -0.5) and Trial Number (within-subject continuous factor, scaled and mean-centred) of each learning session, we calculated an LME model with these factors as fixed effects, expected values as dependent variable and allowed random intercepts by subject, as well as random slopes for Trial Number, Pair Type, and their interaction per participant. Please note that expected values of a pair refer to the expected value of the correct stimulus/action of the pair. Expected values increased across trials, *F*(1,41.01) = 53.55, *p* < .001, *b* = 0.07, and were slightly higher for stimulus pairs than action pairs, *F*(1,41.00) = 9.27, *p* = .004, *b* = 0.03. This effect was further explained by a significant two-way interaction between Pair Type and overall Action Index, *F*(1,41.01) = 73.91, *p* < .001: For participants with lower Action Index values, expected values were higher for stimulus pairs than action pairs (*p* < .001, *b* = 0.11); for participants with higher Action Index values, expected values were higher for action pairs than stimulus pairs (*p* < .001, *b* = -0.05). Finally, a three-way interaction between all factors emerged, *F*(1,40.99) = 7.64, *p* = .008. Resolving the interaction, a two-way interaction between Trial Number and Pair Type emerged only for participants with higher overall Action Index values, *F*(1,41.00) = 4.65, *p* = .037 (*p* = .087 for lower overall Action Index values). These participants increased their expectations throughout the trials only for action pairs, *F*(1,41.00) = 23.04, *p* < .001, *b* = 0.09, but not for stimulus pairs (*p* = .167). All other results were not significant (all *p* ≤ .143).

These results are in line with the previously reported learning rates: participants reliably strengthened expected values across each learning session. As participants with lower Action Index values would select the preferable stimulus more often and participants learned better from positive than negative feedback, expected values would be more pronounced for stimulus pairs, and vice versa for participants with higher Action Index values.

**Prediction Error Values**

We additionally investigated prediction error values across the learning sessions. For a scatter plot of the distribution see Figure S2.C. The figure suggests that while prediction errors got slightly more negative with increasing trials, which is in line with increased expectation values and a respectively increased probability for participants to choose the correct action/stimulus, prediction error values are distributed from -1 to 1 also in later trials.

We conducted a statistical analysis using an LME model with fixed effects Trial Number and overall Action Index, dependent variable prediction error values and random intercepts by participant as well as random slopes for Trial Number by participant. It confirmed that prediction error values decreased slightly across trials, *F*(1,41.01) = 6.83, *p* = .012, *b* = -0.04, but there was no effect of overall Action Index (*p* = .383), and no significant interaction between overall Action Index and Trial Number (*p* = .412).

**Figure S2.C**

*Scatterplot of Prediction Error Values across learning sessions*


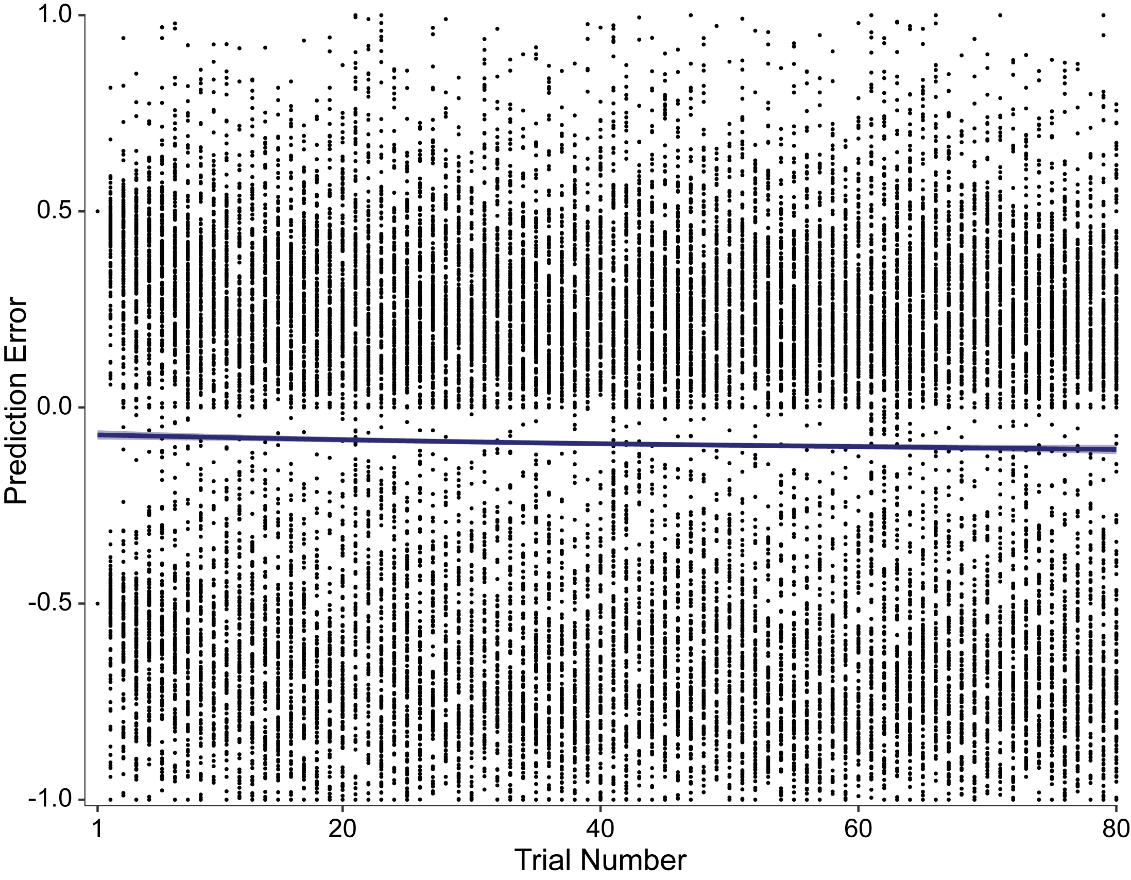


**Section S3**

*Additional investigation of Action Index values by block*

**Figure S3.A**

*Single Subject Action Index Changes by Block*


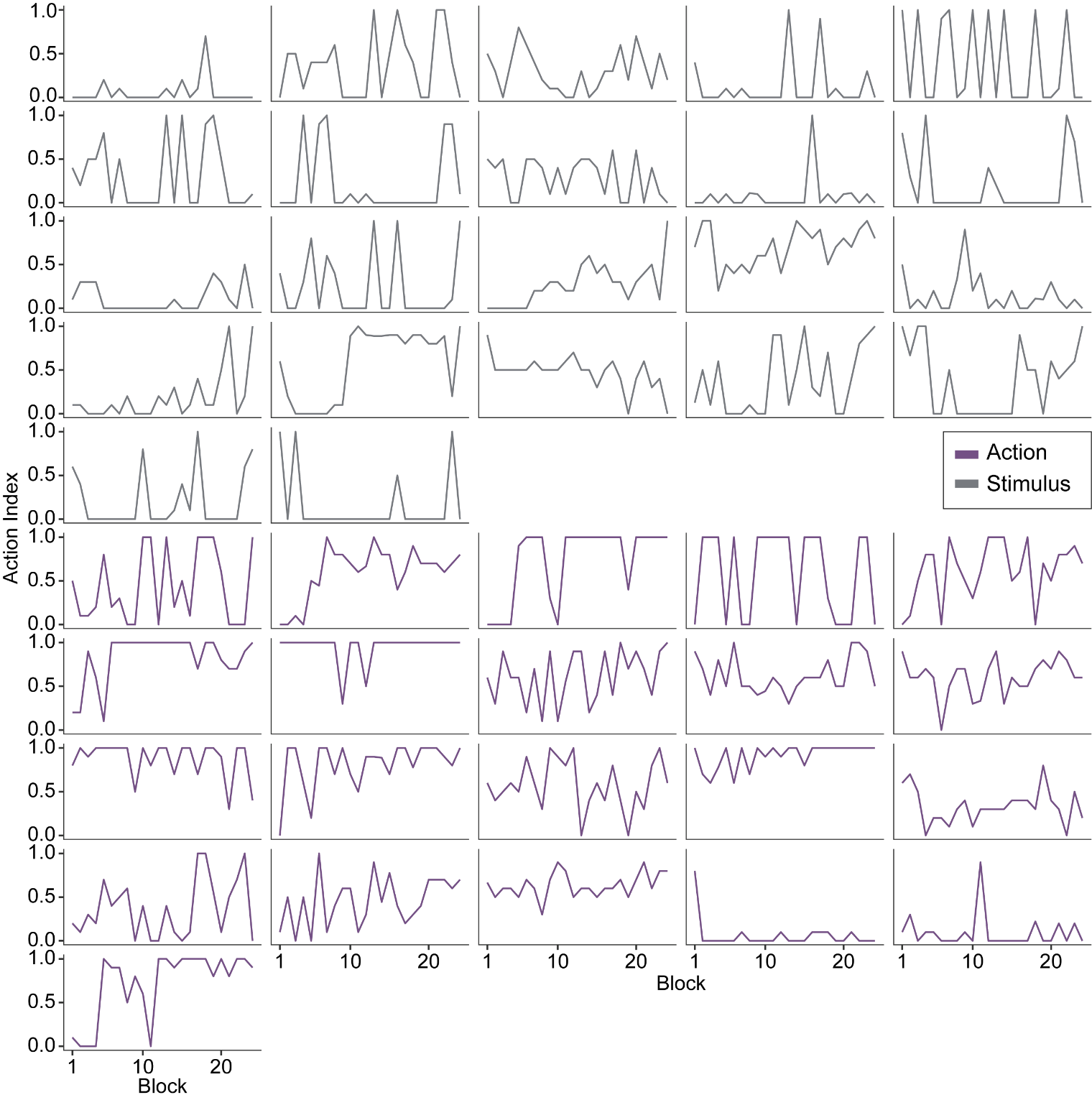


Single subject values of the Action Index changes by block (4 blocks in each of the 6 learning sessions, 24 total) are displayed in Figure S3.A. We quantified two measures of variation: first, variation within participants was calculated for each block as the deviation of the Action Index of this block from the overall Action Index across blocks for this participant. Second, variation between participants was calculated as the deviation of each participant’s overall Action Index across blocks and the mean Action Index of all participants in the respective instruction group. The variation within participants did not differ between the action and stimulus instruction group, *t*(40.45) = 0.87, *p* = .390. The variation between participants within group did also not differ between the action and stimulus instruction group, *t*(34.84) < 0.01, *p* > .999. We also tested an LME model with accuracy in each block as fixed effect and random slope by participant, and deviation from 0.5 of the Action Index of each block as dependent variable. This LME analysis revealed that accuracy predicted the Action Index deviation, *F*(1,34.00) = 36.09, *p* < .001, *b* = 0.17. As expected, lower accuracy meant smaller deviation from 0.5 in the Action Index. However, the intercept for the model was also significant, *t*(41.89) = 22.98, *p* < .001, *b* = 0.36, suggesting that even for low accuracy rates or chance level, the Action Index deviation was different from 0. This result suggests that the Action Index is a valid predictor beyond learning rates. See Figure S3.B for a display of this effect.

**Figure S3.B**

*Linear Effect and Scatterplot on the effect of Accuracy on the deviation from 0.5 of the Action Index*

*
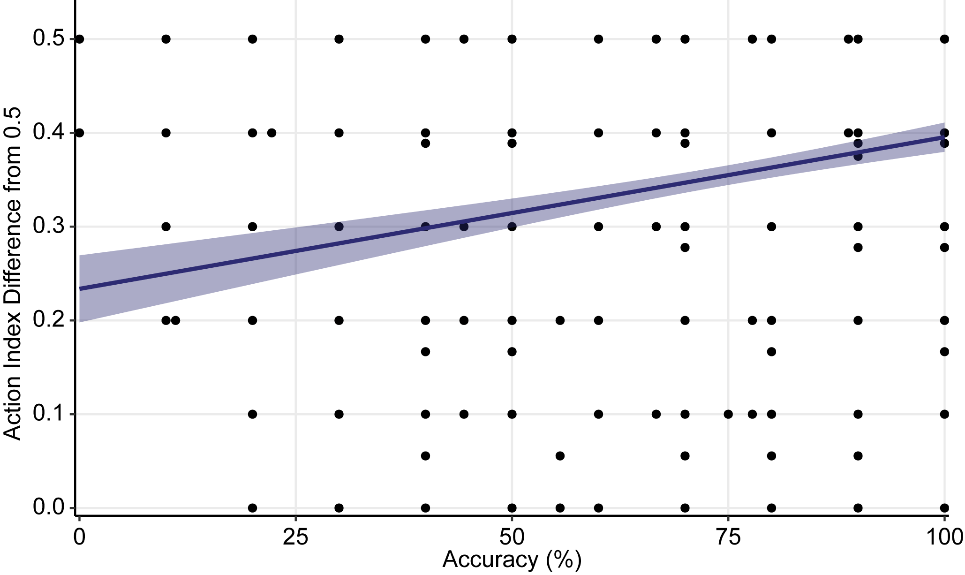
*

*Note.* Single points represent accuracy and Action Index of each block of each participant (24 blocks per participant).

| **Table S1**  *Average latency in ms of negative and preceding positive peaks found in single-subject averages per condition for the FRN/N2* | | | | | | |
| --- | --- | --- | --- | --- | --- | --- |
| Peak | Feedback Timing | Feedback Valence | *M* | *SD* | *Min* | *Max* |
| N2 | Immediate | Negative | 281 | 30 | 227 | 385 |
|  |  | Positive | 303 | 52 | 206 | 392 |
|  | Delayed Feedback Without Tone | Negative | 318 | 49 | 215 | 397 |
|  |  | Positive | 336 | 42 | 216 | 395 |
|  | Delayed Feedback With Tone | Negative | 299 | 37 | 227 | 392 |
|  |  | Positive | 316 | 43 | 207 | 397 |
| P2 | Immediate | Negative | 205 | 24 | 166 | 297 |
|  |  | Positive | 210 | 31 | 126 | 282 |
|  | Delayed Feedback Without Tone | Negative | 208 | 36 | 110 | 281 |
|  |  | Positive | 224 | 36 | 151 | 305 |
|  | Delayed Feedback With Tone | Negative | 198 | 25 | 154 | 266 |
|  |  | Positive | 209 | 25 | 166 | 280 |
| *Note. M* = mean across all participants, *SD* = standard deviation, *Min* = minimum, *Max* = maximum, N2 = maximum negative peak between 200 ms and 400 ms after feedback at pooled signal of Fz, FCz, Cz, FC1 and FC2. P200 = maximum positive peak between 100 ms and the latency of the N2. | | | | | | |
|  |  | | | | | |

| **Table S2**  *Average Latency in ms of negative peaks found in single-subject averages per condition and hemisphere for the N170* | | | | | | |
| --- | --- | --- | --- | --- | --- | --- |
| Elec-trode | Feedback Timing | Feedback Valence | *M* | *SD* | *Min* | *Max* |
| P7 | Immediate | Negative | 206 | 23 | 152 | 247 |
|  |  | Positive | 204 | 17 | 166 | 243 |
|  | Delayed Feedback Without Tone | Negative | 205 | 25 | 151 | 247 |
|  |  | Positive | 204 | 21 | 166 | 245 |
|  | Delayed Feedback With Tone | Negative | 201 | 24 | 151 | 245 |
|  |  | Positive | 202 | 19 | 165 | 242 |
| P8 | Immediate | Negative | 191 | 22 | 145 | 240 |
|  |  | Positive | 202 | 20 | 169 | 244 |
|  | Delayed Feedback Without Tone | Negative | 191 | 25 | 149 | 247 |
|  |  | Positive | 204 | 22 | 161 | 247 |
|  | Delayed Feedback With Tone | Negative | 185 | 20 | 150 | 233 |
|  |  | Positive | 197 | 17 | 162 | 243 |
| *Note. M* = mean across all participants, *SD* = standard deviation, *Min* = minimum, *Max* = maximum. Maximum negative peak is searched between 140 ms and 250 ms after feedback at P7 and P8, respectively. | | | | | | |
|  | | | | | |  |
|  | | | | | |  |

| **Table S3**  *b-values, confidence intervals and t-test results for the GLME analysis on accuracy in the ambiguous trials of the learning trials* | | | | | | |  |
| --- | --- | --- | --- | --- | --- | --- | --- |
| **Effects** | ***b*** | ***SE*** | ***z*** | ***p*** | ***2.5% CI*** | ***97.5% CI*** | |
| Intercept | 1.21 | 0.07 | 16.39 | **< .001** | 1.05 | 1.37 | |
| Instruction | 0.11 | 0.15 | 0.76 | .444 | -0.17 | 0.38 | |
| Delayed Feedback Without Tone | -0.02 | 0.12 | -0.18 | .856 | -0.25 | 0.19 | |
| Delayed Feedback With Tone | -0.05 | 0.13 | -0.38 | .706 | -0.32 | 0.19 | |
| Block | 0.34 | 0.12 | 2.86 | **.004** | 0.07 | 0.58 | |
| Instruction x Delayed Feedback Without Tone | -0.14 | 0.24 | -0.59 | .554 | -0.57 | 0.38 | |
| Instruction x Delayed Feedback With Tone | -0.10 | 0.25 | -0.40 | .686 | -0.65 | 0.39 | |
| Instruction x Block | 0.15 | 0.24 | 0.61 | .541 | -0.38 | 0.58 | |
| Delayed Feedback Without Tone x Block | 0.04 | 0.26 | 0.14 | .889 | -0.50 | 0.54 | |
| Delayed Feedback With Tone x Block | -0.06 | 0.24 | -0.23 | .816 | -0.57 | 0.41 | |
| Instruction x Delayed Feedback Without Tone x Block | 0.74 | 0.50 | 1.48 | .139 | -0.25 | 1.83 | |
| Instruction x Delayed Feedback With Tone x Block | -0.11 | 0.47 | -0.23 | .816 | -1.04 | 0.96 | |
| *Note.* The sign of the *b*-estimates indicates the direction of main effects for the fixed-effects predictors Instruction (action instruction [-0.5] vs. stimulus instruction [0.5]), Feedback Timing (simple coding contrast matrix with immediate feedback set as baseline that is compared with delayed feedback with tone and delayed feedback without tone), and Block (1 [-0.5], 2 [-0.167], 3 [0.167], 4 [0.5]). GLME = generalized linear mixed effects, *b* = beta estimate, *SE* = standard error, *CI* = confidence interval. | | | | | | | |

| **Table S4**  *b-values, confidence intervals and t-test results for the GLME analysis on accuracy in the ambiguous trials of the test trials* | | | | | | |  |
| --- | --- | --- | --- | --- | --- | --- | --- |
| **Effects** | ***b*** | ***SE*** | ***z*** | ***p*** | ***2.5% CI*** | ***97.5% CI*** | |
| Intercept | 1.88 | 0.16 | 11.86 | **< .001** | 1.60 | 2.20 | |
| Instruction | -0.19 | 0.31 | -0.60 | .546 | -0.80 | 0.45 | |
| Delayed Feedback Without Tone | -0.13 | 0.20 | -0.65 | .516 | -0.51 | 0.26 | |
| Delayed Feedback With Tone | -0.18 | 0.18 | -0.95 | .341 | -0.51 | 0.17 | |
| Block | -0.06 | 0.20 | -0.29 | .774 | -0.43 | 0.34 | |
| Instruction x Delayed Feedback Without Tone | -0.33 | 0.38 | -0.86 | .390 | -1.06 | 0.41 | |
| Instruction x Delayed Feedback With Tone | -0.25 | 0.35 | -0.72 | .473 | -0.98 | 0.45 | |
| Instruction x Block | 0.10 | 0.38 | 0.27 | .790 | -0.58 | 0.83 | |
| Delayed Feedback Without Tone x Block | 0.45 | 0.46 | 0.98 | .329 | -0.40 | 1.38 | |
| Delayed Feedback With Tone x Block | -0.46 | 0.49 | -0.93 | .351 | -1.51 | 0.52 | |
| Instruction x Delayed Feedback Without Tone x Block | 1.99 | 0.86 | 2.31 | **.021** | 0.27 | 3.80 | |
| Instruction x Delayed Feedback With Tone x Block | 1.13 | 0.94 | 1.20 | .230 | -0.70 | 3.17 | |
| *Note.* The sign of the *b*-estimates indicates the direction of main effects for the fixed-effects predictors Instruction (action instruction [-0.5] vs. stimulus instruction [0.5]), Feedback Timing (simple coding contrast matrix with immediate feedback set as baseline that is compared with delayed feedback with tone and delayed feedback without tone), and Block (1 [-0.5], 2 [-0.167], 3 [0.167], 4 [0.5]). GLME = generalized linear mixed effects, *b* = beta estimate, *SE* = standard error, *CI* = confidence interval. | | | | | | | |

| **Table S5** |  |  |  |  |  |  |  |
| --- | --- | --- | --- | --- | --- | --- | --- |
| *b-values, confidence intervals and t-test results for the LME analysis on Action Index* | | | | | |  |  |
| **Effects** | ***b*** | ***SE*** | ***df*** | ***t*** | ***p*** | ***2.5% CI*** | ***97.5% CI*** |
| Intercept | 0.43 | 0.03 | 41.00 | 14.02 | **< .001** | 0.37 | 0.49 |
| Instruction | -0.32 | 0.06 | 41.00 | -5.11 | < .**001** | -0.45 | -0.19 |
| Delayed Feedback Without Tone | 0.02 | 0.02 | 979.00 | 0.91 | .364 | -0.03 | 0.07 |
| Delayed Feedback With Tone | -0.04 | 0.02 | 979.00 | -1.60 | .109 | -0.08 | 0.01 |
| Block | -0.01 | 0.03 | 979.00 | -0.25 | .805 | -0.06 | 0.04 |
| Instruction x Delayed Feedback Without Tone | 0.03 | 0.05 | 979.00 | 0.73 | .466 | -0.06 | 0.12 |
| Instruction x Delayed Feedback With Tone | 0.10 | 0.05 | 979.00 | 2.22 | **.027** | 0.00 | 0.19 |
| Instruction x Block | -0.01 | 0.05 | 979.00 | -0.19 | .846 | -0.11 | 0.09 |
| Delayed Feedback Without Tone x Block | -0.05 | 0.06 | 979.00 | -0.86 | .391 | -0.18 | 0.06 |
| Delayed Feedback With Tone x Block | -0.02 | 0.06 | 979.00 | -0.32 | .748 | -0.15 | 0.10 |
| Instruction x Delayed Feedback Without Tone x Block | -0.01 | 0.12 | 979.00 | -0.10 | .918 | -0.23 | 0.22 |
| Instruction x Delayed Feedback With Tone x Block | -0.01 | 0.12 | 979.00 | -0.09 | .931 | -0.27 | 0.22 |
| *Note.* The sign of the *b*-estimates indicates the direction of main effects for the fixed-effects predictors Instruction (action instruction [-0.5] vs. stimulus instruction [0.5]), Feedback Timing (simple coding contrast matrix with immediate feedback set as baseline that is compared with delayed feedback with tone and delayed feedback without tone), and Block (1 [-0.5], 2 [-0.167], 3 [0.167], 4 [0.5]). LME = linear mixed effects, *b* = beta estimate, *SE* = standard error, *df* = degrees of freedom, *CI* = confidence interval. | | | | | | | |

| **Table S6** |  |  |  |  |  |  |  |
| --- | --- | --- | --- | --- | --- | --- | --- |
| *b-values, confidence intervals and t-test results of the LME analysis on the FRN/N2 amplitude including the factor Action Index* | | | | | |  |  |
| **Effects** | ***b*** | ***SE*** | ***df*** | ***t*** | ***p*** | ***2.5% CI*** | ***97.5% CI*** |
| Intercept | -3.67 | 0.28 | 43.00 | -13.31 | **< .001** | -4.19 | -3.11 |
| Action Index | -0.24 | 0.14 | 19172.43 | -1.70 | .089 | -0.56 | 0.05 |
| Delayed Feedback Without Tone | -0.86 | 0.12 | 19305.53 | -7.41 | **< .001** | -1.09 | -0.64 |
| Delayed Feedback With Tone | -1.07 | 0.12 | 19307.52 | -9.34 | **< .001** | -1.33 | -0.87 |
| Feedback Valence | 0.52 | 0.09 | 19306.58 | 5.47 | **< .001** | 0.34 | 0.70 |
| PE | 0.36 | 0.18 | 19306.19 | 2.00 | **.046** | -0.01 | 0.71 |
| Action Index x Delayed Feedback Without Tone | 0.33 | 0.32 | 19317.19 | 1.02 | .306 | -0.27 | 0.95 |
| Action Index x Delayed Feedback With Tone | 0.42 | 0.32 | 19320.96 | 1.31 | .191 | -0.30 | 1.06 |
| Action Index x Feedback Valence | 0.27 | 0.26 | 19305.01 | 1.03 | .302 | -0.29 | 0.81 |
| Delayed Feedback Without Tone x Feedback Valence | -0.74 | 0.23 | 19304.75 | -3.18 | **.001** | -1.22 | -0.31 |
| Delayed Feedback With Tone x Feedback Valence | -0.68 | 0.23 | 19304.98 | -2.95 | **.003** | -1.11 | -0.26 |
| Action Index x PE | -0.01 | 0.46 | 19308.01 | -0.01 | .989 | -0.99 | 0.77 |
| Delayed Feedback Without Tone x PE | 0.25 | 0.44 | 19305.67 | 0.57 | .571 | -0.66 | 1.08 |
| Delayed Feedback With Tone x PE | -0.65 | 0.43 | 19305.33 | -1.48 | .138 | -1.55 | 0.28 |
| Feedback Valence x PE | 1.83 | 0.38 | 19344.90 | 4.85 | **< .001** | 1.12 | 2.58 |
| Action Index x Delayed Feedback Without Tone x Feedback Valence | 1.33 | 0.63 | 19304.60 | 2.10 | **.036** | 0.04 | 2.57 |
| Action Index x Delayed Feedback With Tone x Feedback Valence | 1.63 | 0.63 | 19304.90 | 2.58 | **.010** | 0.38 | 2.87 |
| Action Index x Delayed Feedback Without Tone x PE | 2.25 | 1.12 | 19305.37 | 2.01 | **.045** | 0.12 | 4.48 |
| Action Index x Delayed Feedback With Tone x PE | 1.83 | 1.11 | 19305.33 | 1.64 | .101 | -0.45 | 3.85 |
| Action Index x Feedback Valence x PE | -0.58 | 0.94 | 19343.95 | -0.62 | .537 | -2.34 | 1.16 |
| Delayed Feedback Without Tone x Feedback Valence x PE | 0.10 | 0.88 | 19307.92 | 0.11 | .911 | -1.60 | 1.96 |
| Delayed Feedback With Tone x Feedback Valence x PE | -0.26 | 0.87 | 19308.60 | -0.30 | .761 | -2.32 | 1.44 |
| Action Index x Delayed Feedback Without Tone x Feedback Valence x PE | -1.90 | 2.25 | 19309.94 | -0.84 | .399 | -6.69 | 2.24 |
| Action Index x Delayed Feedback With Tone x Feedback Valence x PE | -1.22 | 2.23 | 19308.91 | -0.54 | .586 | -5.78 | 3.33 |
| *Note*The sign of the *b*-estimates indicates the direction of main effects for the fixed-effects predictors Action Index (scaled and mean centered), Feedback Timing (simple coding contrast matrix with immediate feedback set as baseline that is compared with delayed feedback with tone and delayed feedback without tone), Feedback Valence (negative [-0.5] vs. positive [0.5]) and mean centered unsigned PE (prediction error)*.* LME = linear mixed effects, *b* = beta estimate, *SE* = standard error, *CI* = confidence interval. | | | | | | | |

| **Table S7**  *Number of data points per subject by condition after outlier removal for the FRN/N2 model including Action Index* | | | | |
| --- | --- | --- | --- | --- |
| Feedback Timing | Feedback Valence | *M* | *SD* | *Min* |
| Immediate | Negative | 64.51 | 9.02 | 44 |
|  | Positive | 87.77 | 9.10 | 68 |
| Delayed Feedback Without Tone | Negative | 62.33 | 9.20 | 45 |
|  | Positive | 85.33 | 9.09 | 69 |
| Delayed Feedback With Tone | Negative | 63.84 | 8.12 | 51 |
|  | Positive | 86.67 | 7.70 | 65 |
| *Note. M* = mean across all participants, *SD* = standard deviation, *Min* = minimum. | | | | |
|  | | | | |

| **Table S8** |  |  |  |  |  |  |  |
| --- | --- | --- | --- | --- | --- | --- | --- |
| *b-values, confidence intervals and t-test results of the LME analysis on the N170 amplitude including the factor Action Index* | | | | | | | |
| **Effects** | ***b*** | ***SE*** | ***df*** | ***t*** | ***p*** | ***2.5% CI*** | ***97.5% CI*** |
| Intercept | -6.44 | 0.54 | 42.75 | -12.03 | **< .001** | -7.57 | -5.36 |
| Action Index | 0.21 | 0.13 | 38669.80 | 1.66 | .098 | -0.02 | 0.47 |
| Delayed Feedback Without Tone | -0.92 | 0.10 | 38630.88 | -9.11 | **< .001** | -1.10 | -0.72 |
| Delayed Feedback With Tone | -1.37 | 0.10 | 38631.80 | -13.73 | **< .001** | -1.55 | -1.17 |
| Feedback Valence | 0.88 | 0.08 | 38631.46 | 10.71 | **< .001** | 0.71 | 1.03 |
| PE | -0.07 | 0.15 | 38631.27 | -0.45 | .653 | -0.39 | 0.24 |
| Action Index x Delayed Feedback Without Tone | -0.07 | 0.28 | 38636.35 | -0.25 | .801 | -0.64 | 0.49 |
| Action Index x Delayed Feedback With Tone | -0.12 | 0.28 | 38637.80 | -0.43 | .667 | -0.67 | 0.48 |
| Action Index x Feedback Valence | -0.01 | 0.22 | 38630.81 | -0.03 | .976 | -0.43 | 0.46 |
| Delayed Feedback Without Tone x Feedback Valence | -0.02 | 0.20 | 38630.62 | -0.09 | .930 | -0.41 | 0.41 |
| Delayed Feedback With Tone x Feedback Valence | -0.41 | 0.20 | 38630.81 | -2.05 | **.041** | -0.81 | -0.02 |
| Action Index x PE | 0.00 | 0.40 | 38632.24 | 0.00 | .998 | -0.78 | 0.79 |
| Delayed Feedback Without Tone x PE | 0.23 | 0.38 | 38630.96 | 0.60 | .548 | -0.58 | 0.96 |
| Delayed Feedback With Tone x PE | -0.32 | 0.38 | 38631.04 | -0.83 | .404 | -1.09 | 0.44 |
| Feedback Valence x PE | -1.93 | 0.33 | 38657.84 | -5.89 | **< .001** | -2.57 | -1.28 |
| Action Index x Delayed Feedback Without Tone x Feedback Valence | 0.03 | 0.55 | 38630.66 | 0.05 | .958 | -0.99 | 1.07 |
| Action Index x Delayed Feedback With Tone x Feedback Valence | 0.03 | 0.55 | 38630.82 | 0.05 | .961 | -1.08 | 1.14 |
| Action Index x Delayed Feedback Without Tone x PE | -0.04 | 0.97 | 38630.96 | -0.04 | .965 | -2.08 | 1.85 |
| Action Index x Delayed Feedback With Tone x PE | -0.97 | 0.97 | 38630.98 | -1.00 | .316 | -2.93 | 0.88 |
| Action Index x Feedback Valence x PE | -1.74 | 0.82 | 38654.51 | -2.13 | **.033** | -3.37 | 0.06 |
| Delayed Feedback Without Tone x Feedback Valence x PE | -1.51 | 0.76 | 38632.20 | -1.98 | **.048** | -2.88 | -0.09 |
| Delayed Feedback With Tone x Feedback Valence x PE | -1.29 | 0.76 | 38632.36 | -1.70 | .089 | -2.82 | 0.28 |
| Action Index x Delayed Feedback Without Tone x Feedback Valence x PE | 0.56 | 1.96 | 38633.18 | 0.28 | .776 | -3.15 | 4.41 |
| Action Index x Delayed Feedback With Tone x Feedback Valence x PE | 0.09 | 1.95 | 38632.57 | 0.05 | .963 | -3.64 | 4.34 |
| *Note.* The sign of the *b*-estimates indicates the direction of main effects for the fixed-effects predictors Action Index (scaled and mean centered), Feedback Timing (simple coding contrast matrix with immediate feedback set as baseline that is compared with delayed feedback with tone and delayed feedback without tone), Feedback Valence (negative [-0.5] vs. positive [0.5]) and mean centered unsigned PE (prediction error). LME = linear mixed effects, *b* = beta estimate, *SE* = standard error, *CI* = confidence interval. | | | | | | | |

| **Table S9**  *Number of data points per subject by condition and by electrode after outlier removal for the N170 model including Instruction* | | | | | |
| --- | --- | --- | --- | --- | --- |
| Electrode | Feedback Timing | Feedback Valence | *M* | *SD* | *Min* |
| P7 | Immediate | Negative | 64.35 | 8.36 | 52 |
|  |  | Positive | 87.65 | 9.29 | 68 |
|  | Delayed Feedback Without Tone | Negative | 63.58 | 9.34 | 44 |
|  |  | Positive | 85.60 | 10.27 | 69 |
|  | Delayed Feedback With Tone | Negative | 63.47 | 8.74 | 43 |
|  |  | Positive | 86.30 | 8.08 | 66 |
| P8 | Immediate | Negative | 64.02 | 8.40 | 47 |
|  |  | Positive | 87.49 | 9.35 | 67 |
|  | Delayed Feedback Without Tone | Negative | 62.12 | 8.53 | 44 |
|  |  | Positive | 85.81 | 9.78 | 64 |
|  | Delayed Feedback With Tone | Negative | 63.37 | 9.08 | 44 |
|  |  | Positive | 86.16 | 7.66 | 66 |
| *Note. M* = mean across all participants, *SD* = standard deviation, *Min* = minimum. | | | | | |
|  | | | | | |
|  | | | | | |
